# Stagnation-point flow of a Sisko nanofluid over a stretching surface with heat generation and nano-transport phenomena

**DOI:** 10.1038/s41598-025-31036-8

**Published:** 2025-12-18

**Authors:** Ghulam Muhiuddin, N. Ramya, Farshid Mofidnakhaei, Hossein Rashmanlou, Farah Maqsood, Noura Aldossary

**Affiliations:** 1 Department of Mathematics, Faculty of Science, University of Tabuk, Tabuk, 71491 Saudi Arabia; 2 Department of Mathematics, Kongu Engineering College, Erode, 638060 India; 3 Department of Physics, Sar. C., Islamic Azad University, Sari, Iran; 4 School of Physics, Damghan University, Damghan, 3671645667 Iran; 5 Department of Optometry and Vision Sciences, College of Applied Medical Sciences, King Saud University, Riyadh, 11433 Saudi Arabia

**Keywords:** Sisko nanofluid, Heat generation, Stretching sheet, Stagnation-Point flow, Thermophoresis, Engineering, Mathematics and computing, Nanoscience and technology, Physics

## Abstract

The present investigation focuses on the transient magnetohydrodynamic (MHD) behaviour of the stagnation-point flow of a Sisko nanofluid past a stretching surface, highlighting the interactive influence of internal heat generation and nano-scale transport mechanisms. The study integrates the rheological complexity of the Sisko model with nanoparticle motion induced by Brownian diffusion and thermophoresis, both of which substantially modify the fluid’s momentum, thermal, and solutal layers. Through appropriate similarity transformations, the governing nonlinear partial differential equations are transformed and parameterized into a set of coupled ordinary differential equations, subsequently solved using MATLAB’s bvp4c routine. A systematic evaluation of controlling parameters including the Sisko material constant, Brownian diffusion, thermophoretic strength, and heat generation coefficient—has been performed to elucidate their respective impacts on velocity, temperature, and concentration distributions. The findings reveal that nano-scale diffusion processes intensify both thermal and solutal gradients, whereas internal heat generation augments the thermal boundary layer thickness. This study delivers deeper theoretical understanding of non-Newtonian nanofluid dynamics and underscores its significance for enhanced heat transfer applications in polymer extrusion, coating systems, and advanced thermal manufacturing operations.

## Introduction

The concept of nanofluids has garnered substantial research interest owing to their superior thermal and transport characteristics, which significantly enhance heat transfer performance across numerous engineering and industrial systems. Scholars have extensively examined various nanofluid models, including Sisko and Casson nanofluids, to understand how factors such as magnetic fields, thermal radiation, viscous dissipation, and nanoparticle interactions govern momentum and heat transport processes. Recent studies particularly emphasize the combined influence of Joule heating, viscous dissipation, and magnetic fields on the behaviour of Sisko nanofluids. Hussain et al.^1^ analyzed the MHD flow of Sisko nanofluid over an extending cylinder, highlighting the interplay between viscous dissipation and Joule heating in altering velocity and temperature distributions. Bilal et al.^2^ examined peristaltic motion of Sisko nanofluid under thermal radiation, viscous effects, double-diffusive convection, and a magnetic field, illustrating the coupled influence of these parameters on thermal and solutal transport. Yasmin et al.^3^ explored multiple slip effects in thermally radiative Sisko nanofluid flow, revealing that slip and magnetic parameters significantly affect both temperature and concentration gradients. Nisha and De^4^ investigated Hall and ion slip effects in reactive Sisko nanofluid flow through porous media, noting prominent modifications in flow and heat transfer due to electromagnetic interactions. Almaneea^5^ performed a numerical investigation on ternary Sisko nanofluids, demonstrating how nanoparticle composition and concentration alter effective conductivity and diffusivity. Omama et al.^6^ studied MHD flow of a quadruple hybrid nanofluid through a stenosed artery with porous walls, revealing the dual role of hybrid nanoparticles and magnetic effects in refining flow uniformity and heat transport. Mabood et al.^7^ examined melting and internal heat generation/absorption in Sisko nanofluid flow over a stretching surface, confirming that nonlinear radiation and phase-change processes strongly affect boundary layer thickness and heat transfer intensity. Ijaz et al.^8^ discussed entropy generation and activation energy in nonlinear radiative Sisko nanofluid flow over a rotating disk, emphasizing how electromagnetic parameters enhance system efficiency. Khan et al.^9^ presented a three-dimensional analysis of unsteady MHD Sisko flow with temperature-dependent conductivity, showing that transient and variable properties substantially influence flow structure and heat transport.

Prasannakumara et al.^10^ analyzed nonlinear radiative heat transfer in MHD Sisko nanofluid flow over an extending sheet, illustrating improved heat transfer rates with nonlinear stretching and magnetic field intensity. Eid and Mahny^11^ studied porous media flow of Sisko nanofluid with heat generation/absorption, identifying porosity as a key factor in controlling velocity and temperature fields. Abo-Dahab et al.^12^ investigated double-diffusive peristaltic MHD flow with nonlinear radiation and Joule heating, demonstrating intricate couplings among diffusive and thermal processes. Hayat et al.^13^ focused on stagnation-point flow with melting and internal heating, noting that melting augments heat transfer while heat sources alter boundary layer structure. Al-Mamun et al.^14^ developed predictive models for radiative MHD flow of Sisko nanofluid, underscoring the effect of thermal radiation and magnetic field parameters on thermal efficiency.

Sharma and Bisht^15^ evaluated buoyancy and suction influences on Sisko nanofluid flow in a porous environment, reporting that buoyancy accelerates flow while suction enhances thermal regulation. Bisht and Sharma^16^ numerically examined convective boundary conditions in nonlinear Sisko nanofluid flow, identifying nonlinearity as a major determinant of heat transfer performance. Waseem et al.^17^ applied an enhanced cuckoo search–based optimization algorithm to hybrid Sisko nanofluid flow, demonstrating how machine learning techniques can optimize thermal and flow parameters effectively. In related research, Ramya and Deivanayaki^18^ analyzed Soret and Dufour effects in Casson nanofluid flow under magnetic influence, showing how thermos-diffusion modifies heat and mass transfer. Ramya and Deivanayaki^19^ extended this by simulating Casson micropolar flow through a porous inclined channel, assessing multiple flow and thermal variables. Ramya et al.^20^ investigated radiative Casson nanofluid flow over an inclined surface, confirming the significant role of radiation in enhancing heat transfer. In another work, Ramya et al.^21^ explored thermophoresis and Brownian motion in Casson ternary hybrid nanofluids with bioconvection, revealing complex interactions affecting flow stability and heat transport. Similarly, Khan et al.^22] and [23^ examined thermo-diffusion and radiative effects in Sisko nanofluid flows, establishing how thermal conductivity variations and magnetic influences can be optimized to improve industrial heat management systems.

Mustafa et al.^24^ explored the stagnation-point flow of nanofluids toward a stretching surface, focusing on thermophoretic effects and heat transfer characteristics. Their findings underscored the pivotal role of nanoparticle motion in enhancing both thermal and solutal transport in cooling applications. Ibrahim et al.^25^ examined the MHD stagnation-point flow of a nanofluid toward a stretching sheet, demonstrating how magnetic field strength governs heat transfer enhancement in electromagnetically controlled thermal systems. Bisht and Sharma^26^ examined Sisko nanofluid flow over a stretching cylinder embedded in a porous medium, emphasizing how non-Newtonian behaviour and porosity affect momentum and heat transport. Their analysis revealed that increasing the Sisko index and nanoparticle concentration notably alters velocity gradients and heat transfer efficiency. Munir et al.^27^ investigated the entropy generation characteristics in Oldroyd-B nanofluid stagnation-point flow, emphasizing the influence of viscous dissipation, diffusion irreversibility, and thermal gradients on system efficiency. Khan et al.^28^ explored the impact of thermal radiation and convective heating on hybrid nanofluid stagnation-point flow containing gold and zinc nanoparticles, identifying that radiation enhances heat transfer while altering nodal and saddle-point flow behaviour.

Wang et al.^29^ numerically analyzed the MHD-driven Sisko nanofluid flow with heat and mass transfer, employing a response surface optimization method to quantify the combined roles of magnetic influence, rheological effects, and thermal gradients. Khan et al.^30^ developed a computational model of hybrid Sisko nanofluid flow over a porous, radially heated stretching/shrinking disc, showing that porosity and nanoparticle interactions substantially intensify heat transfer performance. Ramya and Deivanayaki^31^ investigated the Carreau nanofluid flow through a Darcy–Forchheimer porous medium under magnetohydrodynamic conditions, highlighting how bioconvection by motile microorganisms enhances mass transport and flow stability. Raza et al.^32^ performed a finite element study on Boger nanofluids, incorporating morphological effects and nanolayer thermal conductivity under thermal radiation, demonstrating that interfacial nanolayers significantly boost effective heat conduction. Babu et al.^33^ analyzed the squeezed flow of polyethylene glycol–water-based hybrid nanofluid over a magnetized sensor surface, using statistical modelling to evaluate the sensitivity and optimization of heat transfer processes. Deebani et al.^34^ optimized and conducted a sensitivity analysis for tri-hybrid nanofluid stagnation-point flow, concluding that nanoparticle composition and electromagnetic effects are critical in maximizing heat transfer rates. Sahoo and Nandkeolyar^35^ presented an entropy generation analysis of Casson nanofluid stagnation-point flow within a non-Darcy porous medium, incorporating thermal radiation, activation energy, and magnetic induction, and employing a multiple regression framework for prediction accuracy. Tejaswini et al.^36^ studied the combined effects of thermal radiation and thermophoretic deposition in stagnation-point flow over a wavy porous circular cylinder, indicating that surface curvature and porosity markedly enhance particle deposition and heat exchange. Younas and Sagheer^37^ explored nanofluid stagnation-point flow influenced by Brownian motion, thermophoresis, and chemical reaction, revealing that coupled nano-scale transport processes substantially intensify both heat and mass transfer. Ashraf et al.^38^ examined the non-Newtonian nanofluid flow near a stagnation point, demonstrating that thermophoresis and Brownian motion, in conjunction with viscous dissipation, strengthen the coupling between velocity, temperature, and nanoparticle concentration fields^39^. Muhiuddin et al. examined the thermal and bioconvective behaviour of Williamson fluid over a porous curved stretching surface, emphasizing the combined influence of homogeneous–heterogeneous chemical reactions and porous drag on heat, mass, and microorganism transport^40^. Ramya et al. analyzed micropolar nanofluid flow over an exponentially stretching surface incorporating homogeneous–heterogeneous reactions under the Cattaneo–Christov heat flux model, showing that thermal relaxation controls heat transfer while chemical reactions alter solutal concentration distribution. Shaw et al.^41^ formulated the governing model for nonlinear convective Sisko nanofluid flow over an inclined electromagnetic surface considering melting heat transfer effects, whereas Mohanty et al.^42^ advanced the hybrid nanofluid framework by integrating interfacial nanolayer influence, Darcy–Forchheimer drag, and Cattaneo–Christov heat flux to capture improved heat and momentum transport characteristics.

Collectively, these investigations deepen the understanding of nanofluid transport mechanisms and their technological potential in industrial, biomedical, and microfluidic systems. In this context, the present study introduces a novel analysis of Sisko nanofluid flow over a stretching sheet, incorporating internal heat generation and nano-transport phenomena such as Brownian motion and thermophoresis. Unlike previous steady or Newtonian models, this work addresses time-dependent effects within a non-Newtonian Sisko framework, thereby providing a more physically realistic portrayal of heat and mass transfer processes. The integration of temporal stretching and internal heat source/sink mechanisms enhances model accuracy, while the adoption of nano-transport theory bridges a critical gap in existing non-Newtonian thermal transport research. The present research introduces a distinctive advancement by formulating an MHD Casson micropolar tri-hybrid nanofluid model over a porous curved stretching surface, integrating thermal radiation, bioconvection, and homogeneous–heterogeneous chemical reactions in a unified framework. In contrast to prior works that investigated these mechanisms separately or under steady-state conditions, this study uniquely examines their simultaneous nonlinear interactions to capture the complex coupling between momentum, heat, and mass transport phenomena. This comprehensive approach establishes a novel theoretical framework that broadens the understanding of non-Newtonian nanofluid behaviour and provides new predictive insights applicable to energy, biomedical, and material processing systems.

## Mathematical formulation

The proposed model establishes a two-dimensional steady-state boundary layer formulation for a Sisko non-Newtonian nanofluid, capturing multiple coupled transport mechanisms.

**Fig. 1:**
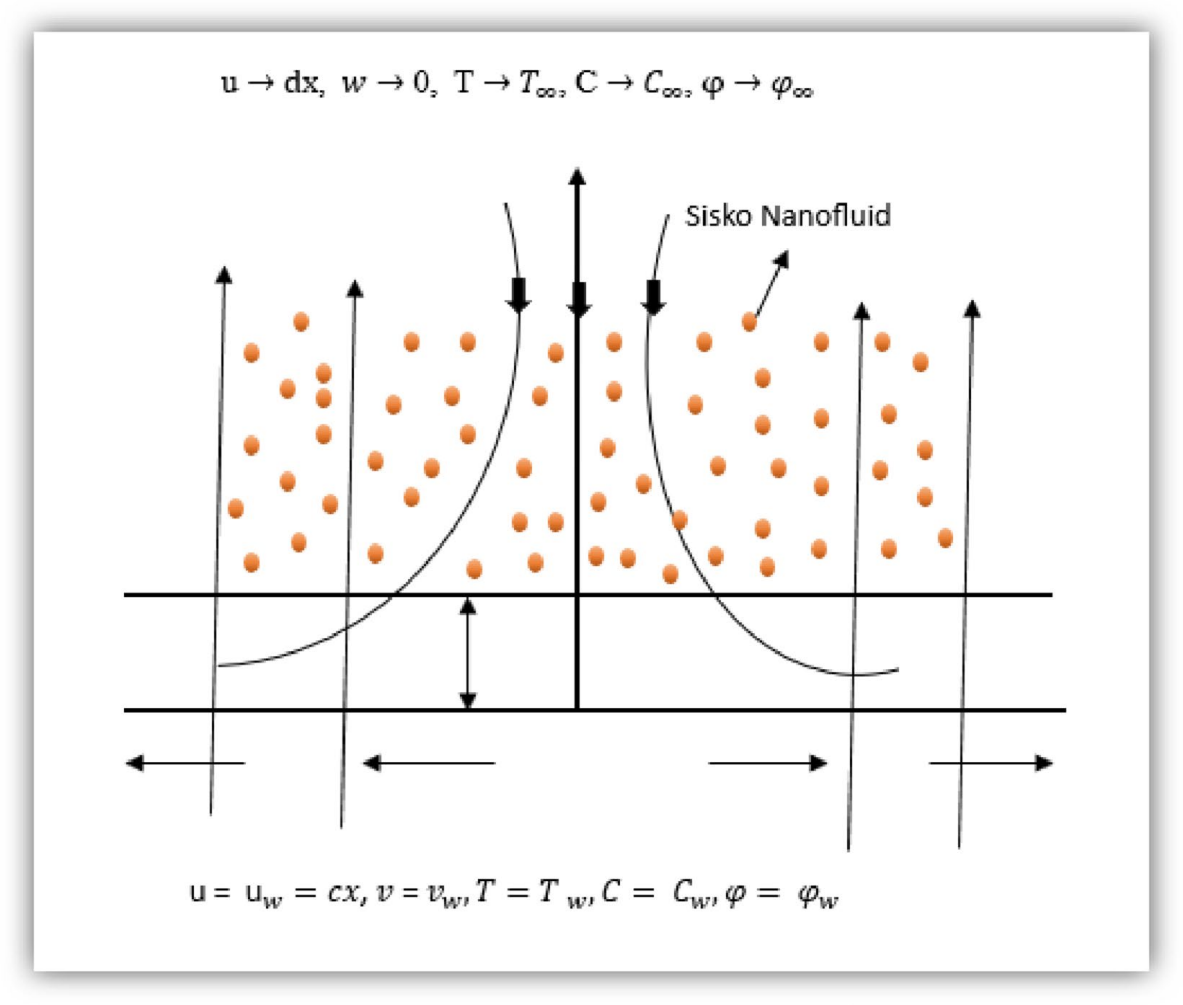
Flow Representation.

It systematically incorporates the influences of a transverse magnetic field (MHD effects), convective heat and mass transfer, Brownian motion, thermophoretic diffusion, and chemical reactions within the flow domain. The governing equations of momentum, energy, and concentration are developed by considering the nonlinear, shear-dependent viscosity intrinsic to the Sisko fluid—defined by the power-law index (n), which dictates whether the fluid demonstrates shear-thinning (*n* < 1) or shear-thickening (*n* > 1) characteristics. Further refinement is achieved through the inclusion of Joule heating, viscous dissipation, and nonlinear radiative heat flux, which enhance the model’s physical accuracy. These combined effects illustrate the strong coupling among velocity, temperature, and concentration profiles in magneto-thermofluidic systems. Collectively, the formulation establishes a robust mathematical framework for analysing flow dynamics, heat transport, and mass diffusion in Sisko nanofluids subjected to multiple thermophysical interactions. This approach provides a theoretically comprehensive foundation for optimizing thermal management and industrial fluid systems involving complex non-Newtonian behaviour, as schematically represented in Fig. 1. Shaw et al.^41^ and Mohanty et al.^42^.

1 $$\:\frac{\partial\:u}{\partial\:x}\mathrm{\:\:+\:\:}\frac{\partial\:v}{\partial\:y}=0$$

2 $$\:u\frac{\partial\:u}{\partial\:x}\mathrm{\:\:+\:\:}\:v\frac{\partial\:u}{\partial\:y}\mathrm{\:\:=\:}{U}_{\infty\:}\frac{d{U}_{\infty\:}}{dx}+\frac{a}{{\rho\:}_{f}}\left(\frac{{\partial\:}^{2}u}{\partial\:{y}^{2}}\right)-\frac{b}{{\rho\:}_{f}}\frac{\partial\:}{\partial\:y}{\left[-\frac{\partial\:u}{\partial\:y}\right]}^{n}+\frac{\sigma\:{{B}_{0}}^{2}}{{\rho\:}_{f}}\left({U}_{\infty\:}-u\right)\:$$

3 $$\:u\frac{\partial\:T}{\partial\:x}+v\frac{\partial\:T}{\partial\:y}={\alpha\:}_{m}\frac{{d}^{2}T}{d{y}^{2}}+\frac{\sigma\:{B}_{0}^{2}}{{\left(\rho\:{C}_{p}\right)}_{f}}{({U}_{\infty\:}-u)}^{2}+\frac{1}{{\left(\rho\:{C}_{p}\right)}_{f}}[a{\left(\frac{\partial\:u}{\partial\:y}\right)}^{2}+b{(-\frac{\partial\:u}{\partial\:y})}^{n+1}]+\frac{\tau\:{D}_{B}}{{\left(\rho\:{C}_{p}\right)}_{f}}\left(\frac{\partial\:\phi\:}{\partial\:y}\frac{\partial\:T}{\partial\:y}\right)+\frac{{D}_{CT}}{{\left(\rho\:{C}_{p}\right)}_{f}}\frac{{\partial\:}^{2}C}{\partial\:{y}^{2}}+\frac{{Q}_{0}}{{\left(\rho\:{C}_{p}\right)}_{f}}$$

4 $$\:u\frac{\partial\:C}{\partial\:x}+v\frac{\partial\:C}{\partial\:y}={D}_{S}\frac{{\partial\:}^{2}C}{\partial\:{y}^{2}}+{D}_{TC}\frac{{\partial\:}^{2}T}{\partial\:{y}^{2}}$$

5 $$\:u\frac{\partial\:\phi\:}{\partial\:x}+v\frac{\partial\:\phi\:}{\partial\:y}={D}_{B}\frac{{\partial\:}^{2}\phi\:}{\partial\:{y}^{2}}+\frac{{D}_{T}}{{T}_{\infty\:}}\frac{{\partial\:}^{2}T}{\partial\:{y}^{2}}$$

$$\:\mathrm{u\:=\:\:}{\mathrm{u}}_{w}=cx\mathrm{,\:}v\:\mathrm{=\:}{v}_{w},\:T={T\:}_{w},\:C=\:{C}_{w},\:\phi\:=\:{\phi\:}_{w}\:\:\mathrm{a}\mathrm{s}\:y=0$$

6 $${\mathrm{u}}\to {\mathrm{dx}}\text{, } w\to \text{0, } {\mathrm{T}}\to {T}_{\infty }\text{, C}\to {C}_{\infty }\varphi\to {\varphi }_{\infty }\:{\mathrm{As}}\:y\to\:\infty\:$$

The boundary conditions define the behaviour of the nanofluid at the surface of the extensive sheet and far away from it in the free stream. At the wall (i.e., when $\:y=0$), the horizontal velocity $\:u$ is equal to a stretching velocity$\:\:{\mathrm{u}}_{w}=cx$, and the vertical velocity v equals a prescribed wall velocity$\:\:{v}_{w}$. Additionally, the temperature T, solutal concentration C, and tiny particle concentration H are all fixed at specific wall values $\:{T\:}_{w}$​, $\:{C}_{w}$​, and $\varphi_{w}$​, respectively, representing a controlled thermal and solutal environment at the surface. As the distance from the wall increases and approaches the free stream ($\:y\to\:\infty\:$), the fluid’s horizontal velocity $\:u$ tends toward a linear free-stream velocity $\:ax$, the vertical velocity $\:v$ diminishes to zero, and the temperature, solutal concentration, and nanoparticle concentration gradually approach their respective ambient values $\:{T}_{\infty\:}\mathrm{,\:}{C}_{\infty\:}$,​, and $\varphi_{\infty\:}$​. These conditions ensure that the influence of the wall decays smoothly and the system transitions to undisturbed ambient flow and thermal states.

$$\:u={\mathrm{u}}_{w}{f}^{{\prime\:}}\left(\eta\:\right),$$

$$\:\nu\:=c{\left(\frac{{c}^{n-2}}{\raisebox{1ex}{${\rho\:}_{f}$}\!\left/\:\!\raisebox{-1ex}{$b$}\right.}\right)}^{\raisebox{1ex}{$1$}\!\left/\:\!\raisebox{-1ex}{$\left(n+1\right)$}\right.}\left(\frac{2}{n+1}f+\frac{1-n}{1+n}\eta\:{f}^{{\prime\:}}\right){x}^{\raisebox{1ex}{$\left(1-n\right)$}\!\left/\:\!\raisebox{-1ex}{$\left(1+n\right)$}\right.},$$

$$\:\:\eta\:=y{\left(\frac{{c}^{2-n}}{\raisebox{1ex}{$b$}\!\left/\:\!\raisebox{-1ex}{${\rho\:}_{f}$}\right.}\right)}^{\raisebox{1ex}{$1$}\!\left/\:\!\raisebox{-1ex}{$\left(n+1\right)$}\right.}{x}^{\raisebox{1ex}{$\left(1-n\right)$}\!\left/\:\!\raisebox{-1ex}{$\left(1+n\right)$}\right.},\:$$

$$\:\theta\:\left(\eta\:\right)=\left[\frac{\left(T-{T}_{\infty\:}\right)}{{T}_{w}-{T}_{\infty\:}}\right],\:$$

$$\:S\left(\eta\:\right)=\left[\frac{C-{C}_{\infty\:}}{{C}_{w}-{C}_{\infty\:}}\right],$$

7 $$\:\phi\:\left(\eta\:\right)=\left[\frac{\varphi-\varphi_{\infty\:}}{\varphi_{w}-\varphi_{\infty\:}}\right]$$

8 $$\:A{f}^{{\prime\:}{\prime\:}{\prime\:}}+n{\left({-f}^{{\prime\:}{\prime\:}}\right)}^{n-1}{f}^{{\prime\:}{\prime\:}{\prime\:}}+\frac{2n}{n+1}{f}^{{\prime\:}}{f}^{{\prime\:}{\prime\:}}+\frac{{d}^{2}}{{c}^{2}}-{f}^{{\prime\:}2}+M\left(\frac{d}{c}-{f}^{{\prime\:}}\right)=0$$

9 $$\:{\theta\:}^{{\prime\:}{\prime\:}}+Pr\frac{2n}{n+1}f{\theta\:}^{{\prime\:}}+PrMEc{\left(1-{f}^{{\prime\:}}\right)}^{2}+PrEc\left(A{\left({f}^{{\prime\:}{\prime\:}}\right)}^{2}+{\left(-{f}^{{\prime\:}{\prime\:}}\right)}^{n+1}\right)+PrNb{\theta\:}^{{\prime\:}}{\phi\:}^{{\prime\:}}+PrNt{\theta\:}^{{\prime\:}2}+Nd{S}^{{\prime\:}{\prime\:}}=0$$

10 $$\:{S}^{{\prime\:}{\prime\:}}+LePr\frac{2n}{n+1}f{S}^{{\prime\:}}+Ld{\theta\:}^{{\prime\:}{\prime\:}}=0$$

11 $$\:\varphi\:{\prime\:}{\prime\:}+LbPr\frac{2n}{n+1}f{\phi\:}^{{\prime\:}}+\frac{Nt}{Nb}{\theta\:}^{{\prime\:}{\prime\:}}=0$$

By introducing affinity transformations, the governing partial differential equations for impulse, energy, solute, and nanoparticle transport are reduced to ordinary differential equations. The velocity field is expressed in terms of a similarity function, while the effective viscosity and similarity variable depend on fluid properties and the flow’s non-Newtonian characteristics. The temperature, solute concentration, and nanoparticle concentration are scaled to dimensionless profiles relative to their ambient and wall values. The momentum equation incorporates both linear and nonlinear shear terms, along with magnetic field effects. The energy equation includes conductive heat transfer, magnetic (Joule) heating, viscous dissipation, and energy contributions from Brownian and thermophoretic diffusion. The concentration equations capture the effects of solutal and nanoparticle diffusion along with thermal cross-diffusion influences.

$$\:f\left(0\right)={f}_{w},\:{f}^{{\prime\:}}\left(0\right)=1,\:\theta\:\left(0\right)=1,\mathrm{S}\left(0\right)=1,\:{\phi\:}\left(0\right)=1\:$$

12 $$\:\:\:{\mathrm{f}}^{{\prime\:}}\left({\infty\:}\right)\to\:\raisebox{1ex}{$\mathrm{d}$}\!\left/\:\!\raisebox{-1ex}{$c$}\right.,\:\:{\uptheta\:}\left({\infty\:}\right)\to\:0,\mathrm{S}\left({\infty\:}\right)\to\:0,\:\:{\phi\:}\left({\infty\:}\right)\to\:0$$

Boundary conditions at the wall include specified stretching velocity and fixed values for temperature and concentrations, while at infinity, the flow variables asymptotically match free-stream values. The system is characterized by several dimensionless parameters such as the magnetic parameter, Prandtl number, Brownian and thermophoretic diffusion parameters, and energy dissipation ratio all of which significantly affect the fluid behaviour within the boundary layer.

13 $$\begin{aligned}\:&A=\frac{R{{e}_{b}}^{\frac{2}{\left(n+1\right)}}}{R{e}_{a}},\:M=\frac{\sigma\:{{B}_{0}}^{2}}{{\rho\:}_{f}c},Pr=\frac{\mu\:{\left({C}_{p}\right)}_{f}}{{k}_{f}},\:Nb=\frac{{\left(\rho\:C\right)}_{f}{\mathrm{D}}_{B}\left({C}_{w}-{C}_{\infty\:}\right)}{{\alpha\:}_{m}{\left(\rho\:C\right)}_{f}},\:Nt=\frac{{\left(\rho\:C\right)}_{f}{\mathrm{D}}_{T}\left({T}_{w}-{T}_{\infty\:}\right)}{{\alpha\:}_{m{T}_{\infty\:}}{\left(\rho\:C\right)}_{f}},\:Le=\frac{{\alpha\:}_{m}}{{\mathrm{D}}_{S}},\:\:\\&Lb=\frac{{\alpha\:}_{m}}{{\mathrm{D}}_{B}},\:Nd=\frac{{D}_{TC}\left({C}_{w}-{C}_{\infty\:}\right)}{{\alpha\:}_{m}\left({T}_{w}-{T}_{\infty\:}\right)},\:Ld=\frac{{D}_{CT}\left({T}_{w}-{T}_{\infty\:}\right)}{{\alpha\:}_{m}\left({C}_{w}-{C}_{\infty\:}\right)},\:s=-\frac{n+1}{2n}\frac{{v}_{w}}{{\left(\frac{{c}^{n-2}}{\raisebox{1ex}{${\rho\:}_{f}$}\!\left/\:\!\raisebox{-1ex}{$b$}\right.}\right)}^{\raisebox{1ex}{$1$}\!\left/\:\!\raisebox{-1ex}{$\left(n+1\right)$}\right.}},\:Ec=\frac{{{u}_{w}}^{2}}{{c}_{p}\left({T}_{w}-{T}_{\infty\:}\right)}\end{aligned}$$

In the context of non-Newtonian fluid models, the power-law index n is a critical parameter that defines the fluid’s rheological behaviour:

- When $\:n=1$, the liquid exhibits Newtonian exploit, meaning the shear stress varies linearly with the shear rate.
- For $\:n>1$, the liquid becomes strain-rate-dependent thickening its viscosity increases with increasing shear rate. As a result, the fluid resists flow more strongly, dominant to a reduction in velocity as $\:n$ increases. This also correlates with reduced sheet slenderness, as the fluid resists deformation more.
- Conversely, when $\:n<1$, the fluid behaves as shear-thinning, where viscosity decreases with higher shear rates, allowing the fluid to flow more readily under stress.

Thus, the power-law index $\:n$ significantly influences the flow characteristics. Higher values of $\:n\:$correspond to increased flow resistance and reduced fluid velocity, while lower values facilitate easier flow.

The important engineering parameters for this flow problem are the skin-friction coefficient, the local Nusselt number, the local Sherwood number and the local nanoparticle Sherwood number. They quantify the wall shear stress, heat transfer, solute mass transfer, and nanoparticle mass transfer at the stretching surface, respectively.

$$\:{C}_{fx}=\frac{{(a+b|\frac{\partial\:u}{\partial\:y}\left|\right)}^{n-1}}{\frac{1}{2}{\rho\:}_{f}{u}_{w}^{2}}{\left(\frac{\partial\:u}{\partial\:y}\right)}_{y=0},$$

$$\:N{u}_{x}=\frac{-x{\left(\frac{\partial\:u}{\partial\:y}\right)}_{y=0}}{({T}_{w}-{T}_{\infty\:})},$$

$$\:S{h}_{x}=\frac{-x{\left(\frac{\partial\:C}{\partial\:y}\right)}_{y=0}}{({C}_{w}-{C}_{\infty\:})},$$

$$\:S{h}_{x,n}=\frac{-x{\left(\frac{\partial\:{\upvarphi\:}}{\partial\:y}\right)}_{y=0}}{({{\upvarphi\:}}_{w}-{{\upvarphi\:}}_{\infty\:})}$$

For the Sisko non-Newtonian model, two characteristic Reynolds numbers appear due to the combined Newtonian and non-Newtonian viscosity terms:

$$\:R{e}_{a}=x{u}_{w}{\rho\:}_{f}/a\:\mathrm{a}\mathrm{n}\mathrm{d}\:R{e}_{b}={x}^{n}{\left({u}_{w}\right)}^{n-2}{\rho\:}_{f}/b$$

These parameters quantify the relative importance of inertial to viscous effects for the linear (a) and nonlinear (b) components of the Sisko fluid, respectively. Using the similarity transformations and nondimensional variables defined earlier, the above dimensional quantities are rewritten as fully dimensionless expressions:

$$\:R{e}_{b}^{\frac{1}{n+1}}{C}_{fx}=A{F}^{{\prime\:\prime\:}}\left(0\right)-{(-{F}^{{\prime\:\prime\:}}(0\left)\right)}^{n},$$

$$\:R{e}_{b}^{\frac{-1}{n+1}}N{u}_{x}=-{\theta\:}^{{\prime\:}}\left(0\right),$$

$$\:R{e}_{b}^{\frac{-1}{n+1}}S{h}_{x}=-{S}^{{\prime\:}}\left(0\right),$$

$$\:R{e}_{b}^{\frac{-1}{n+1}}S{h}_{x,n}=-{{\phi\:}}^{{\prime\:}}\left(0\right).$$

For simplicity in graphical and tabular results, these expressions are typically written in compact dimensionless forms as:

14 $$\:{C}_{f}=R{{e}_{b}}^{\frac{1}{n+1}}{C}_{fx}=A{f}^{{\prime\:}{\prime\:}}\left(0\right)-{\left(-{f}^{{\prime\:}{\prime\:}}\left(0\right)\right)}^{n}$$

15 $$\:Nu=R{{e}_{b}}^{\frac{-1}{n+1}}{Nu}_{x}=-{\theta\:}^{{\prime\:}}\left(0\right)$$

16 $$\:Sh=R{{e}_{b}}^{\frac{-1}{n+1}}{Sh}_{x}=-{S}^{{\prime\:}}\left(0\right)$$

17 $$\:S{h}_{n}=R{{e}_{b}}^{\frac{-1}{n+1}}{Sh}_{xn}=-{\varphi\:}^{{\prime\:}}\left(0\right)$$

Where $\:{C}_{f}$ represents the dimensionless wall shear stress, which increases with higher magnetic or viscosity parameters due to stronger flow resistance. $\:N\mathrm{u}\:$denotes the dimensionless heat transfer rate, inversely related to the thermal boundary layer thickness. $\:Sh$ quantifies the solute mass transfer rate, influenced by diffusion coefficients and cross-diffusion (Soret) effects. $\:S{h}_{n}$ measures the nanoparticle concentration gradient at the wall, reflecting the effects of Brownian motion and thermophoresis. The provided Eqs. 14–17 describe the dimensionless coefficients for friction, warmth deportation, mass deportation, and tiny particle mass deportation in the boundary layer flow of a non-Newtonian tiny liquid. The friction coefficient is determined by the Reynolds number and is influenced by the velocity gradient at the wall, incorporating a non-Newtonian factor. Similarly, the warmth deportation coefficient is related to the Reynolds number and is derived from the temperature gradient at the wall. The mass deportation coefficient, which measures the diffusion of solutes, is also linked to the Reynolds number and the solute concentration gradient at the wall. Finally, the nanoparticle mass transfer coefficient, which quantifies the transfer of nanoparticles, is expressed similarly, depending on the Reynolds number and the nanoparticle concentration gradient at the wall. These coefficients help assess the relative importance of different transfer processes in the fluid flow system.

### Solution methodology

**Fig. 2:**
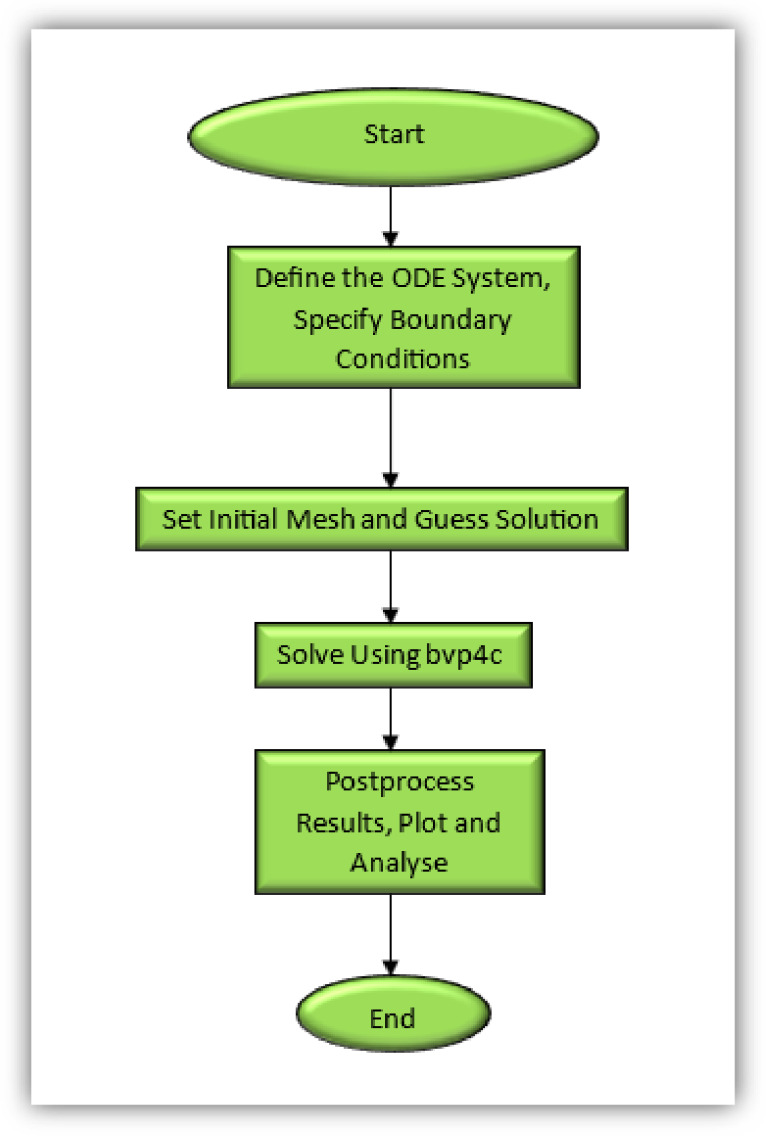
Mechanism of the Bvp4c scheme.

The coupled nonlinear ordinary differential Eqs. (8)-(11) describing the Sisko nanofluid flow are highly nonlinear and therefore do not admit analytical solutions. To obtain accurate results, the system is solved numerically using the MATLAB built-in boundary value problem solver bvp4c. The method transforms the boundary value problem into a system of algebraic equations using adaptive mesh refinement, guaranteeing convergence by maintaining the residual error below a prescribed tolerance. The overall workflow used to resolve the boundary value problem through the Bvp4c algorithm is illustrated in Fig. 2, which depicts the fundamental operational structure of the method.

To facilitate the numerical computation, the higher-order nonlinear differential equations are transformed into a system of first-order equations by introducing the following new variables:

$$\:{y}_{1}=f,\:{y}_{2}={f}^{{\prime\:}},\:{y}_{3}={f}^{{\prime\:}{\prime\:}},\:{y}_{4}=\theta\:,\:{y}_{5}={\theta\:}^{{\prime\:}},\:{y}_{6}=S,\:{y}_{7}={S}^{{\prime\:}},\:{y}_{8}=\varphi\:,\:{y}_{9}={\varphi\:}^{{\prime\:}}$$

The derivatives are therefore given by:

$$\:{y}_{1}^{{\prime\:}}={y}_{2},\:{y}_{2}^{{\prime\:}}={y}_{3}$$

Using Eqs. (8)-(11), the governing equations are reduced to the following first-order system:

$$\:{\mathrm{y}}_{3}^{{\prime\:}}=\frac{{y}_{2}^{2}-\left(2n/n+1\right){y}_{1}{y}_{3}-\left({d}^{2}/{c}^{2}\right)-M\left(d/c-{y}_{2}\right)}{A+n{\left(-{y}_{3}\right)}^{n-1}}$$

$$\:{y}_{5}^{{\prime\:}}=-\mathrm{P}\mathrm{r}\frac{2n}{n+1}{y}_{1}{y}_{5}-\mathrm{P}\mathrm{r}MEc{\left(1-{y}_{2}\right)}^{2}-\mathrm{P}\mathrm{r}Ec\left[A{\left({y}_{3}\right)}^{2}+{\left(-{y}_{3}\right)}^{n+1}\right]-\mathrm{P}\mathrm{r}Nb{y}_{5}{y}_{9}-\mathrm{P}\mathrm{r}Nt{y}_{5}^{2}-Nd{y}_{7}^{{\prime\:}}$$

$$\:{y}_{7}^{{\prime\:}}=-Le\mathrm{P}\mathrm{r}\frac{2n}{n+1}{y}_{1}{y}_{7}-Ld{y}_{5}^{{\prime\:}}$$

$$\:{y}_{9}^{{\prime\:}}=-Lb\mathrm{P}\mathrm{r}\frac{2n}{n+1}{y}_{1}{y}_{9}-\frac{Nt}{Nb}{y}_{5}^{{\prime\:}}$$

The corresponding boundary conditions for the system are defined as:

$$\:At\:\eta\:=0:f=0,\:{f}^{{\prime\:}}=1,\:\theta\:=1,\:S=1,\:\varphi\:=1,As\:\eta\:\to\:{\infty\:}:{f}^{{\prime\:}}\to\:0,\:\theta\:\to\:0,\:S\to\:0,\:\varphi\:\to\:0.$$

The bvp4c solver is implemented with an adaptive mesh containing 10,000 equally spaced points initially, which are automatically refined during the iteration process until the maximum residual error falls below the tolerance level of $\:{10}^{-7}$. The convergence of the numerical results is verified by varying the mesh density and comparing the output values of $\:{f}^{{\prime\:}{\prime\:}}\left(0\right),{\theta\:}^{{\prime\:}}\left(0\right)$, and $\:{\varphi\:}^{{\prime\:}}\left(0\right)$, which correspond to the local skin friction coefficient, Nusselt number, and Sherwood number, respectively. The solutions remain stable and grid-independent, confirming the reliability of the adopted numerical scheme. The present numerical results have been validated by comparing with previously published data available in the literature for limiting cases of Newtonian and power-law fluids. The excellent agreement obtained demonstrates the accuracy and robustness of the implemented method.

## Result and discussion

**Fig. 3:**
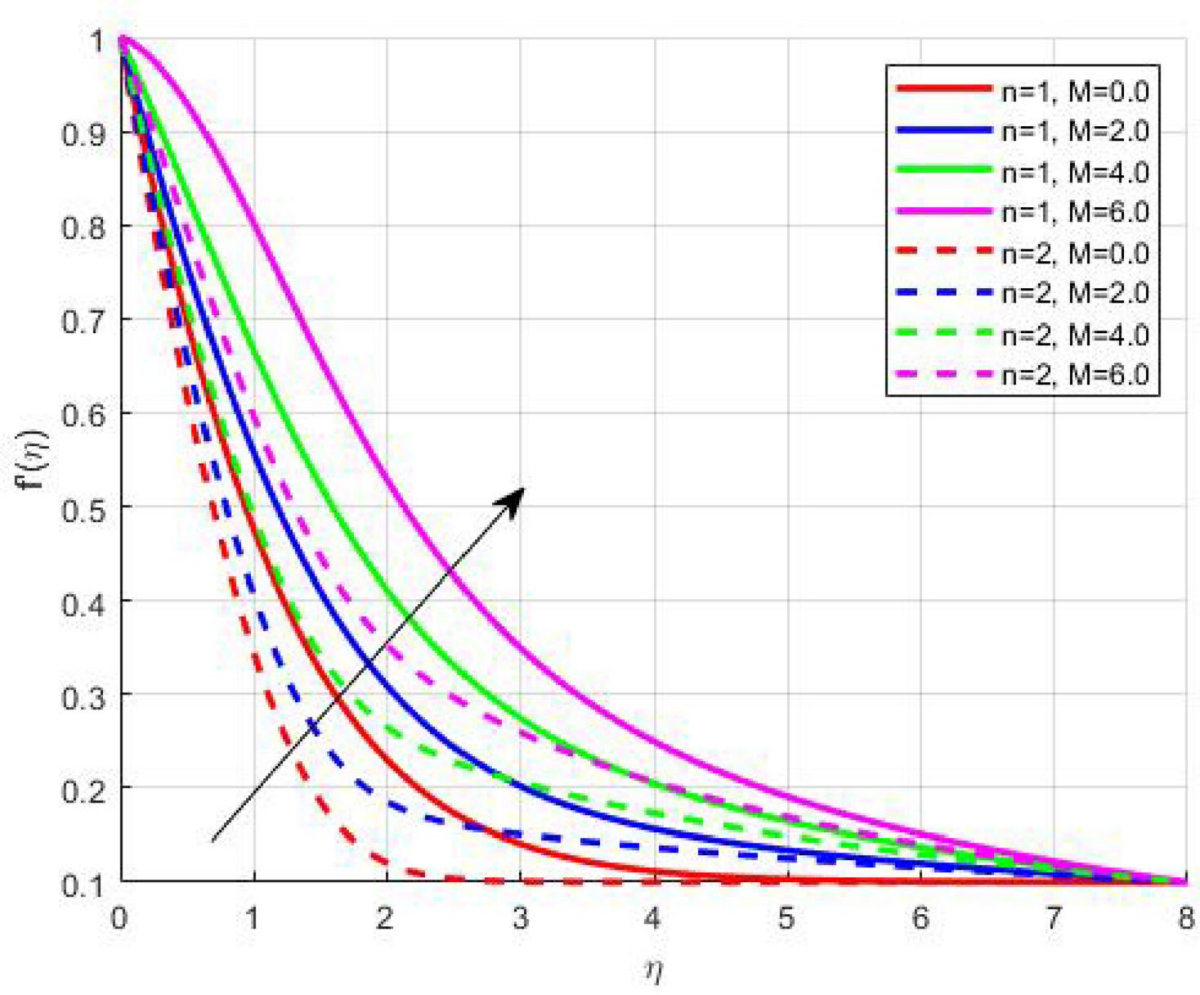
$$\:{f}^{{\prime\:}}\left(\eta\:\right)$$ on M.

**Fig. 4:**
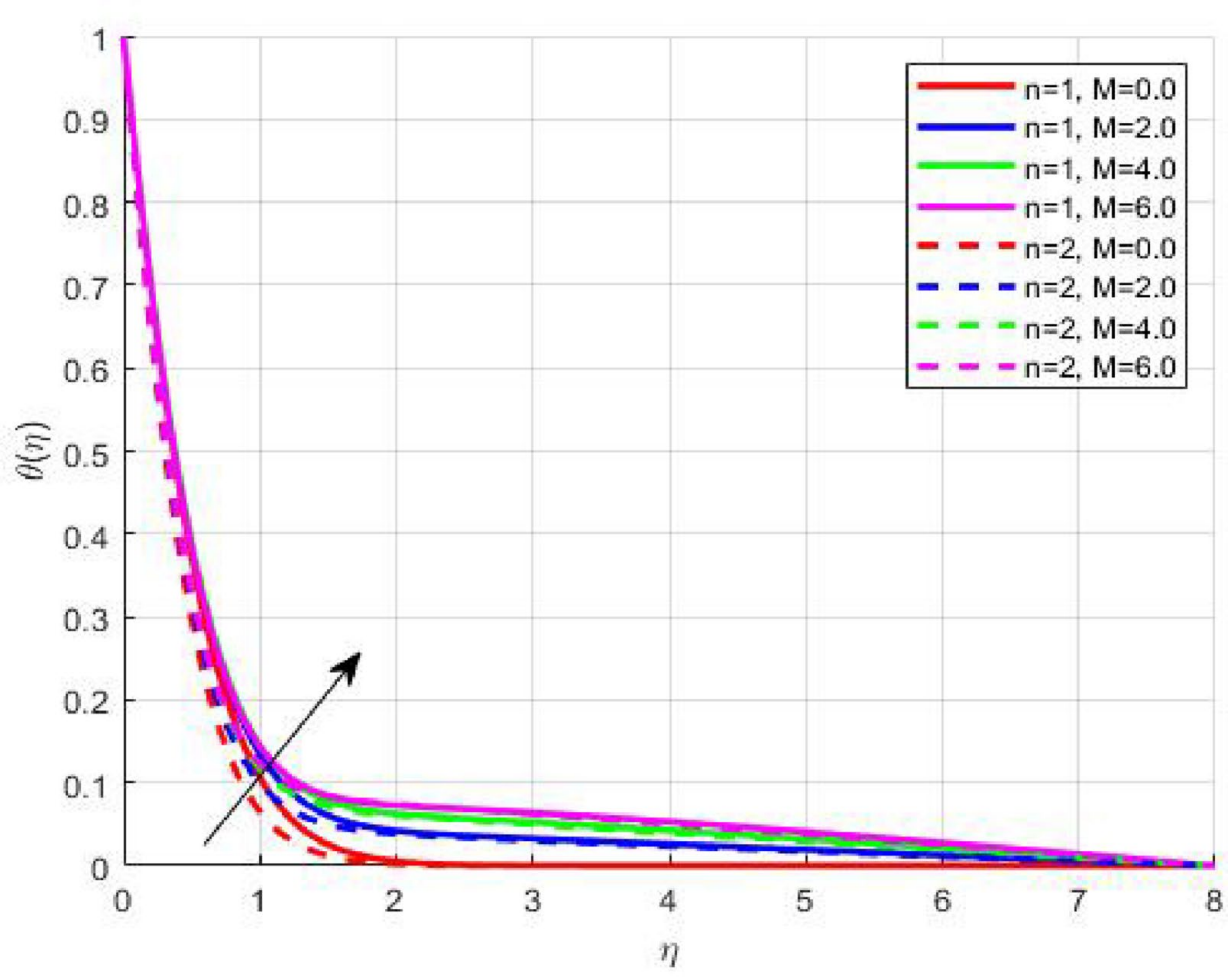
$$\:\theta\:\left(\eta\:\right)$$ on $$\:M$$.

**Fig. 5:**
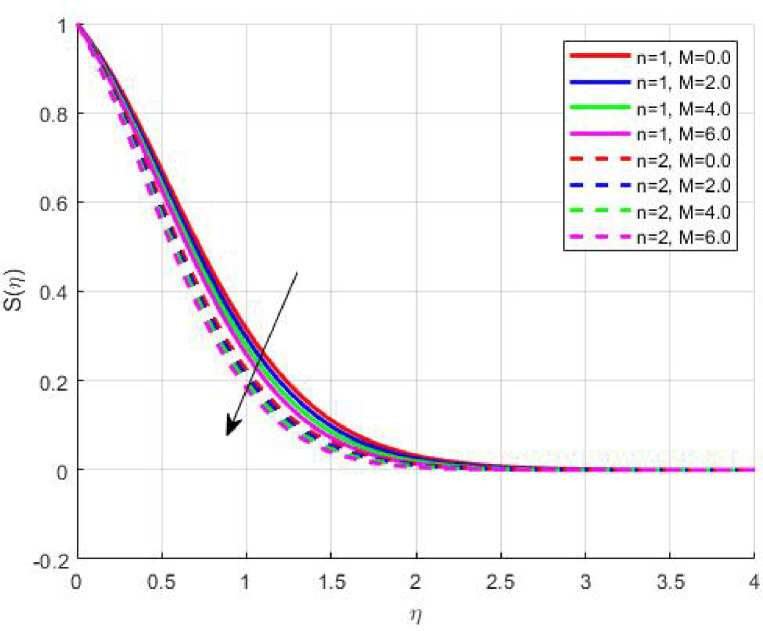
$$\:S\left(\eta\:\right)$$ on $$\:M$$.

**Fig. 6:**
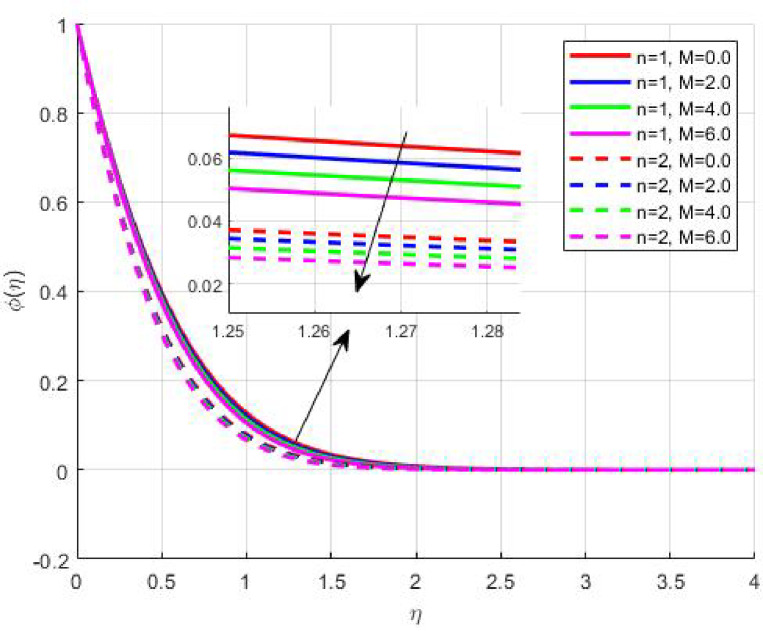
$$\:\phi\:\left(\eta\:\right)$$ on $$\:M$$.

**Fig. 7:**
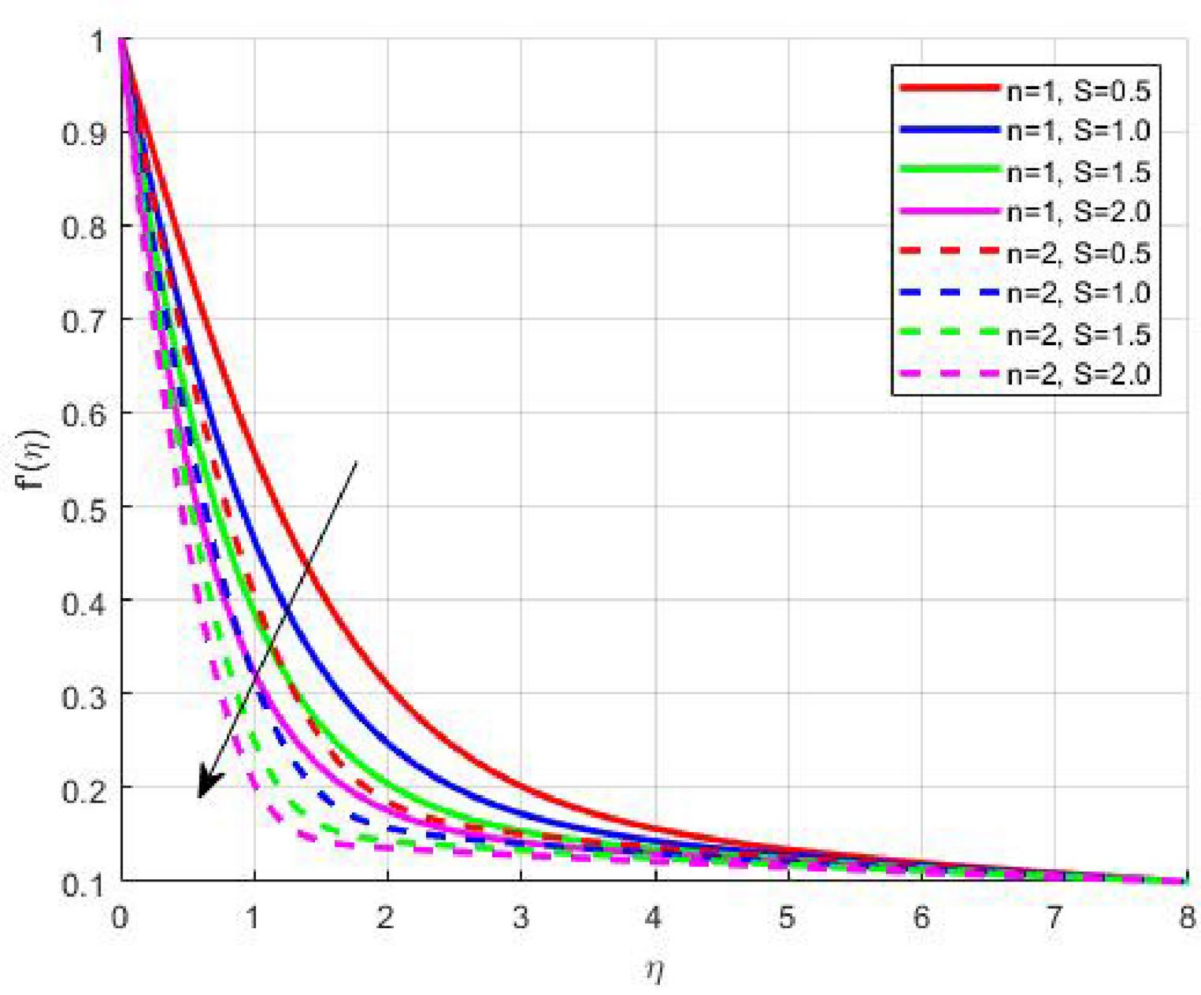
$$\:{f}^{{\prime\:}}\left(\eta\:\right)\:\mathrm{o}\mathrm{n}\:S>0$$

**Fig. 8:**
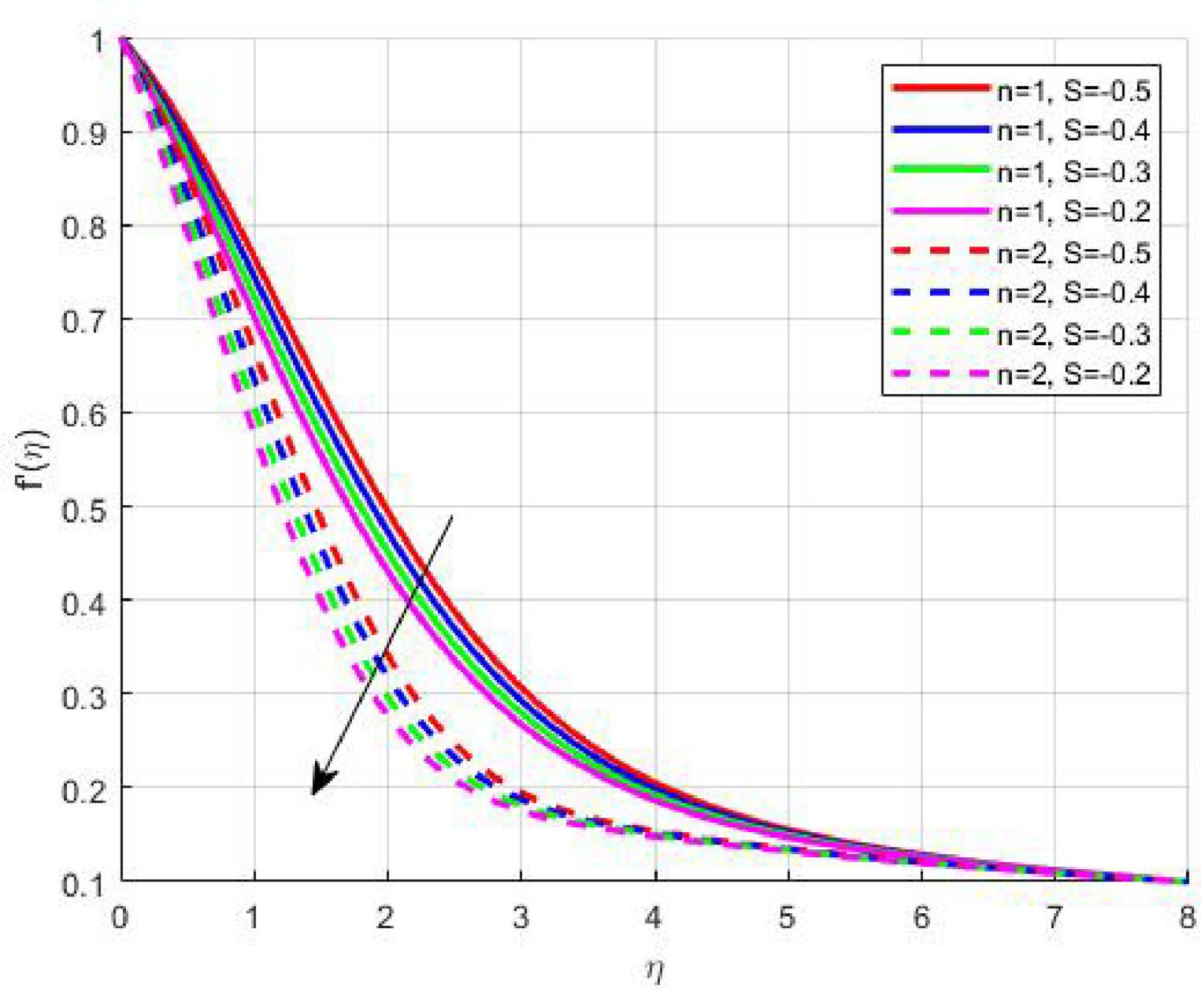
$$\:{f}^{{\prime\:}}\left(\eta\:\right)\:\mathrm{o}\mathrm{n}\:S<0$$

**Fig. 9:**
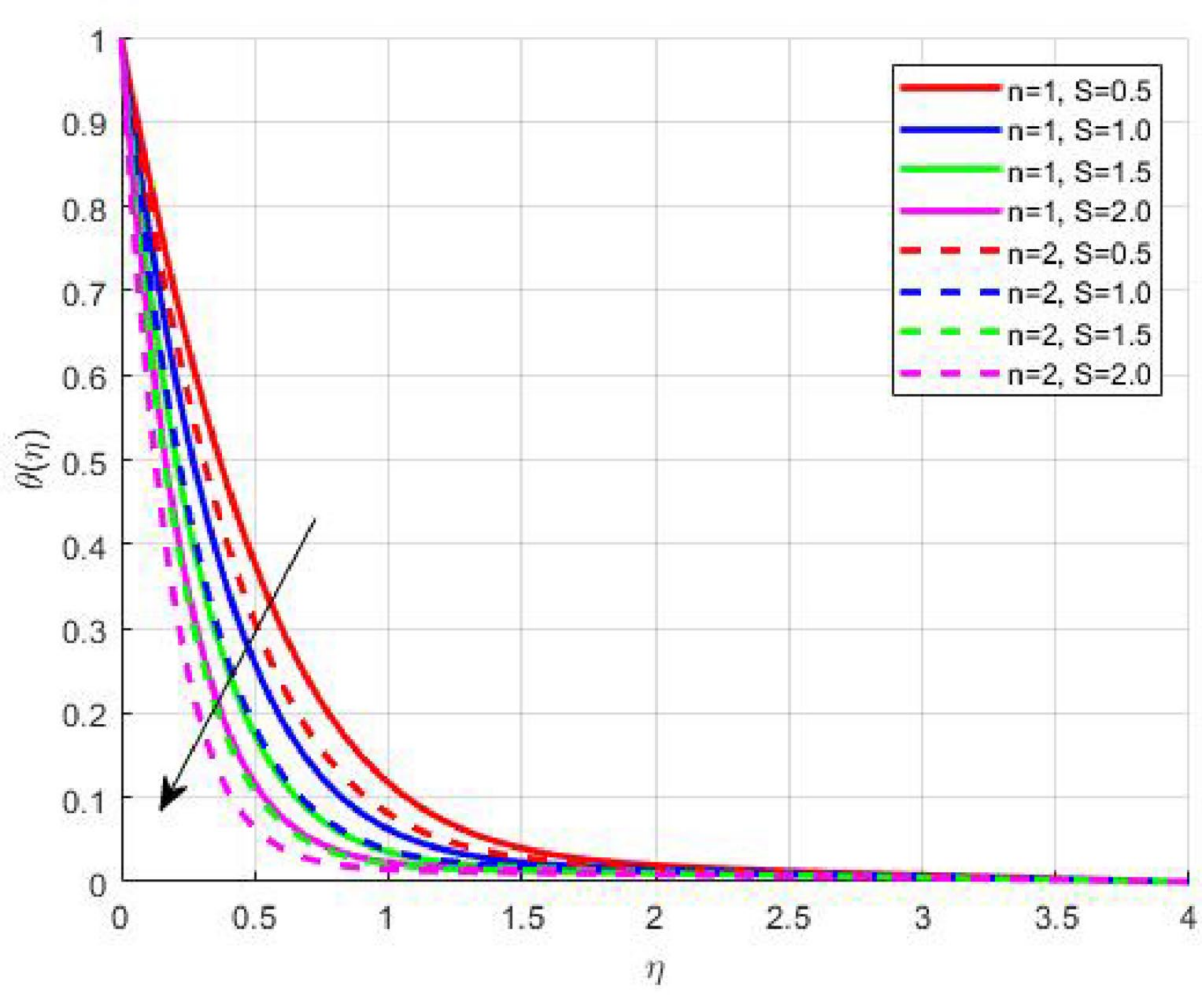
$$\:\theta\:\left(\eta\:\right)\:\mathrm{o}\mathrm{n}\:S>0$$

**Fig. 10:**
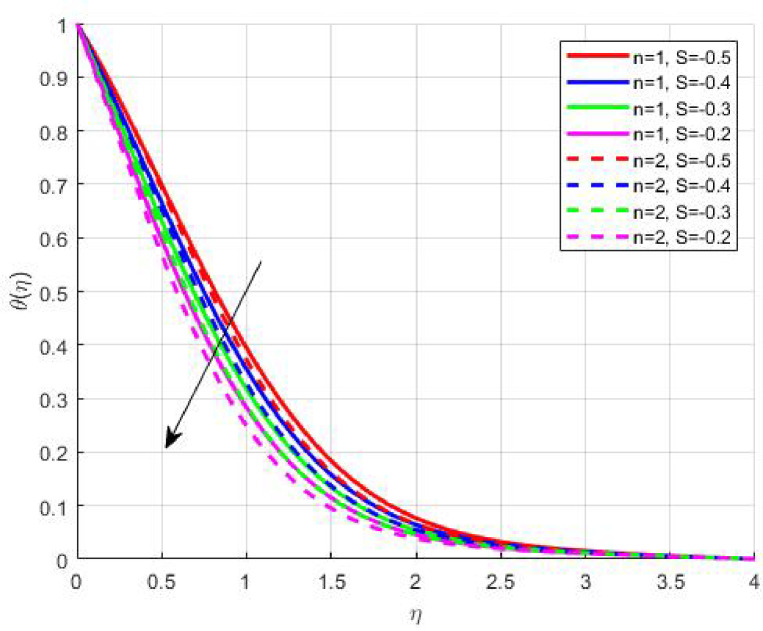
$$\:\theta\:\left(\eta\:\right)\:\mathrm{o}\mathrm{n}\:S<0$$

The Results and Discussion section provides a detailed interpretation of the simulated outcomes, emphasizing the physical mechanisms that control the flow, heat, and mass transfer behaviour of Sisko nanofluids under diverse operating conditions. The numerical findings explore how variations in suction, magnetic field strength, Prandtl number, Lewis number, thermophoretic and Brownian motion parameters modify velocity, temperature, solutal, and nanoparticle concentration profiles. These parameters collectively dictate the nanofluid’s transport efficiency, which is crucial for engineering processes such as microchannel cooling, chemical processing, energy conversion, and coating technologies. Figures 3, 4, 5 and 6 demonstrate the influence of suction and magnetic field intensity on velocity profiles for two different power-law indices (*n* = 1 and *n* = 2). An increase in suction parameter substantially diminishes the velocity magnitude throughout the boundary layer. Physically, stronger suction extracts fluid from the boundary layer toward the wall, thereby weakening the momentum transport and compressing the hydrodynamic layer. The presence of a magnetic field further amplifies this reduction due to the Lorentz force acting opposite to the fluid motion. This electromagnetic resistance converts part of the kinetic energy into magnetic energy, leading to pronounced velocity suppression. The combined effect of suction and magnetization thus provides a powerful mechanism to modulate flow intensity, particularly useful in applications requiring drag reduction, thermal control, or stabilization of conducting fluids. Figures 7, 8, 9 and 10 highlight the behaviour of the temperature field under varying suction levels. For both positive (*S > 0*) and negative (*S < 0*) suction, the temperature profiles decline as suction intensifies. Physically, higher suction enhances convective heat removal by drawing thermal energy away from the wall, thereby reducing the thickness of the thermal boundary layer. The magnetic field also plays a cooling role by resisting fluid motion, which decreases the rate of viscous heating within the fluid. Consequently, the temperature depression observed reflects a synergistic thermal regulation mechanism controlled by suction and electromagnetic damping. This combined control is advantageous for advanced thermal management systems, such as electronic cooling or metallurgical solidification processes.

**Fig. 11:**
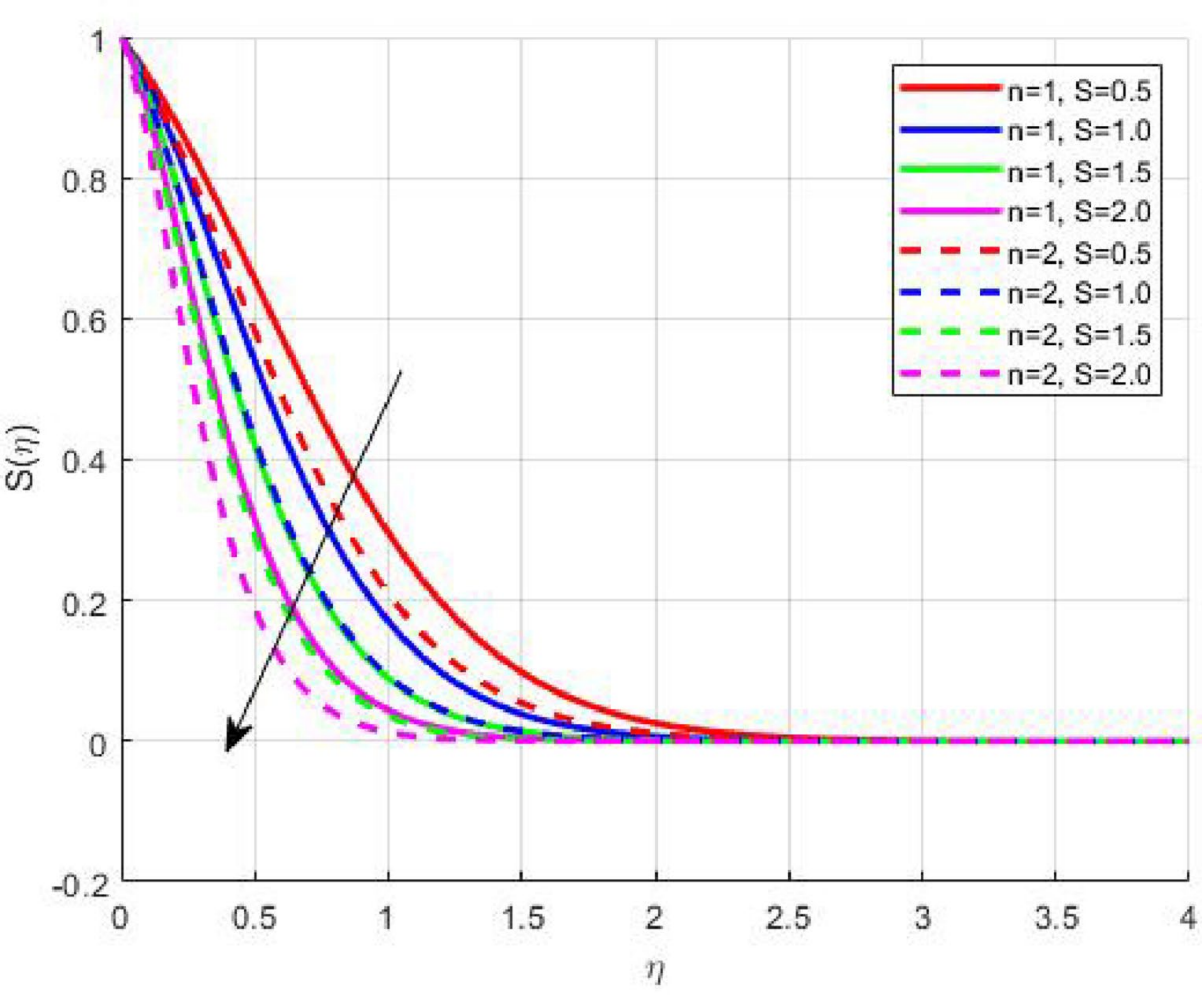
$$\:S\left(\eta\:\right)\:\mathrm{o}\mathrm{n}\:S>0$$

**Fig. 12:**
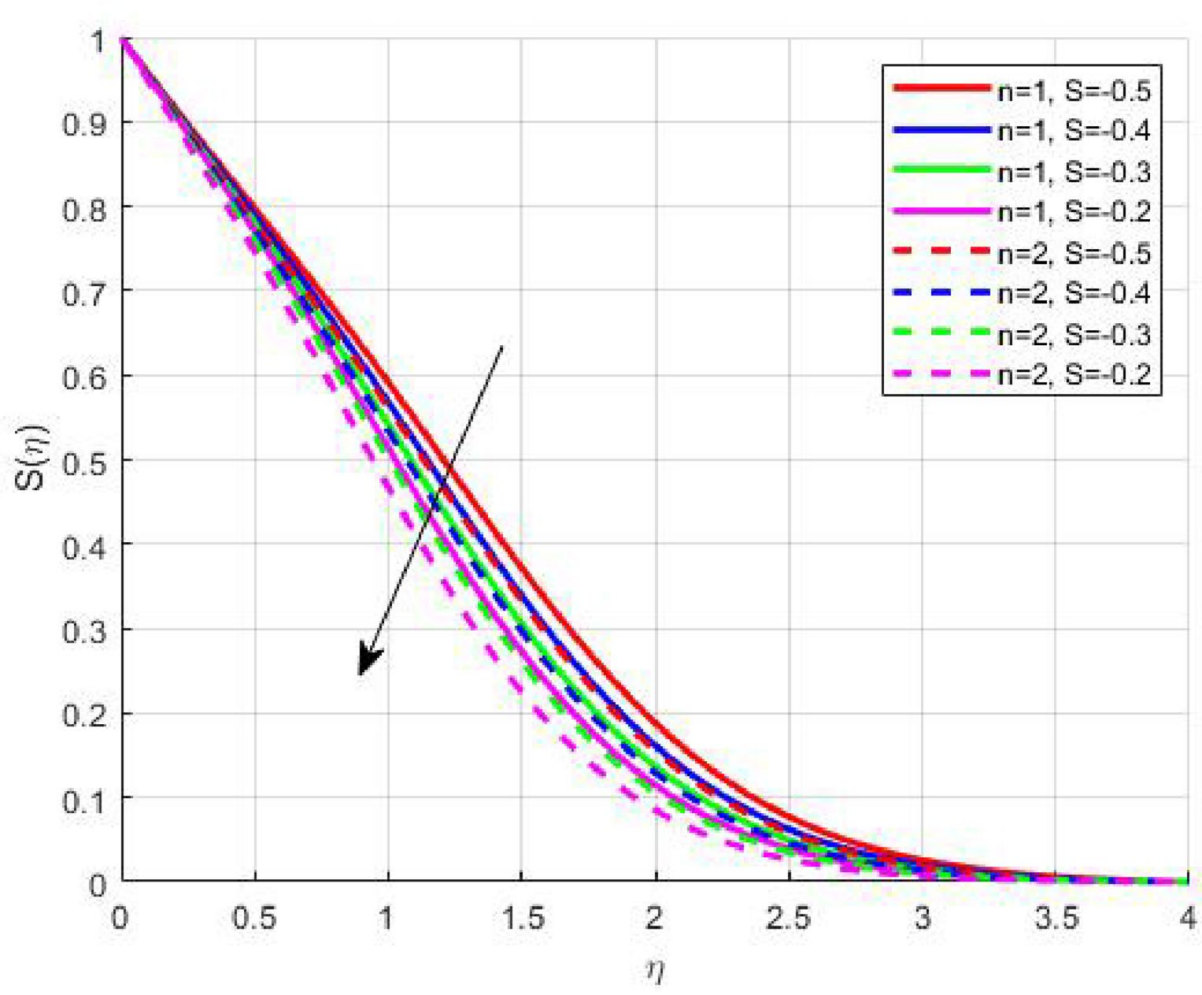
$$\:S\left(\eta\:\right)\:\mathrm{o}\mathrm{n}\:S<0$$

**Fig. 13:**
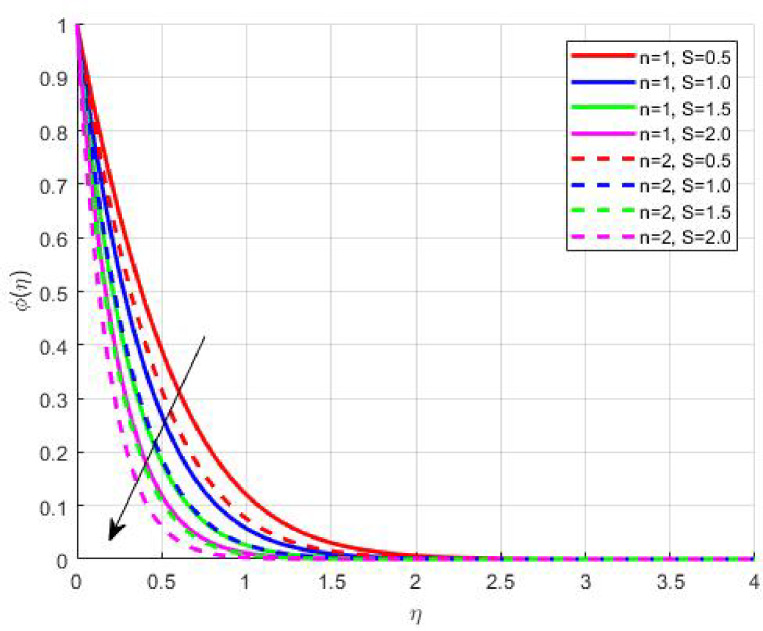
$$\:\phi\:\left(\eta\:\right)\:\mathrm{o}\mathrm{n}\:S>0$$

**Fig. 14:**
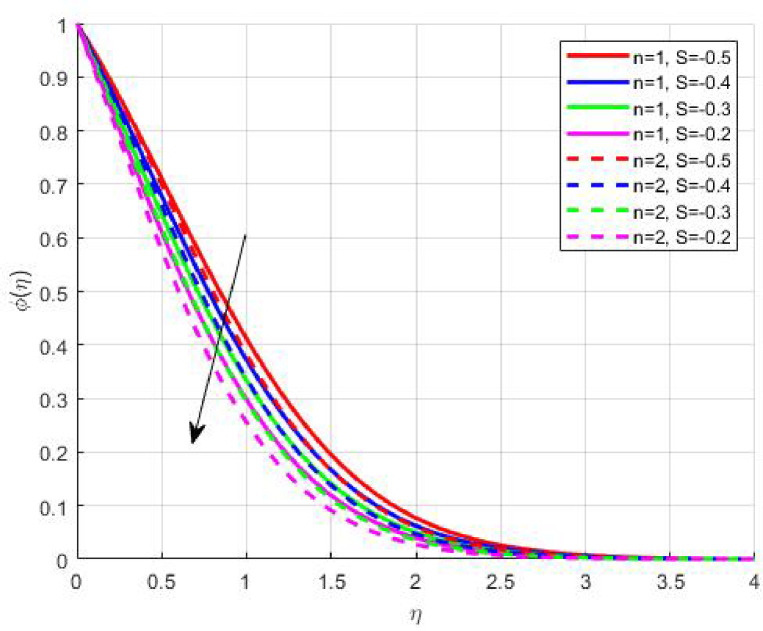
$$\:\phi\:\left(\eta\:\right)\:\mathrm{o}\mathrm{n}\:S<0$$

Figures 11, 12, 13 and 14 elucidate the variations in solutal and nanoparticle concentration with respect to suction. Both profiles exhibit a declining trend with increasing suction strength. From a physical perspective, suction reduces the residence time of nanoparticles and solutes near the wall, promoting their convective removal and attenuating the diffusive flux toward the surface. As a result, concentration gradients weaken, and the solutal as well as nanoparticle boundary layers become thinner. This observation underscores the role of suction in mitigating accumulation phenomena, which is particularly relevant in filtration, corrosion prevention, and chemical vapor deposition processes where surface purity and uniform concentration profiles are desired.

**Fig. 15:**
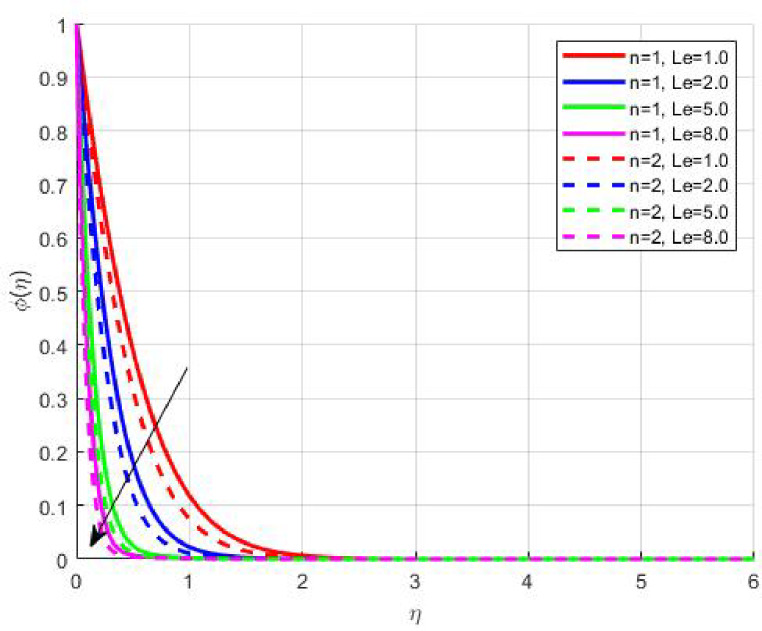
$$\:\phi\:\left(\eta\:\right)$$ on $$\:Le$$.

**Fig. 16:**
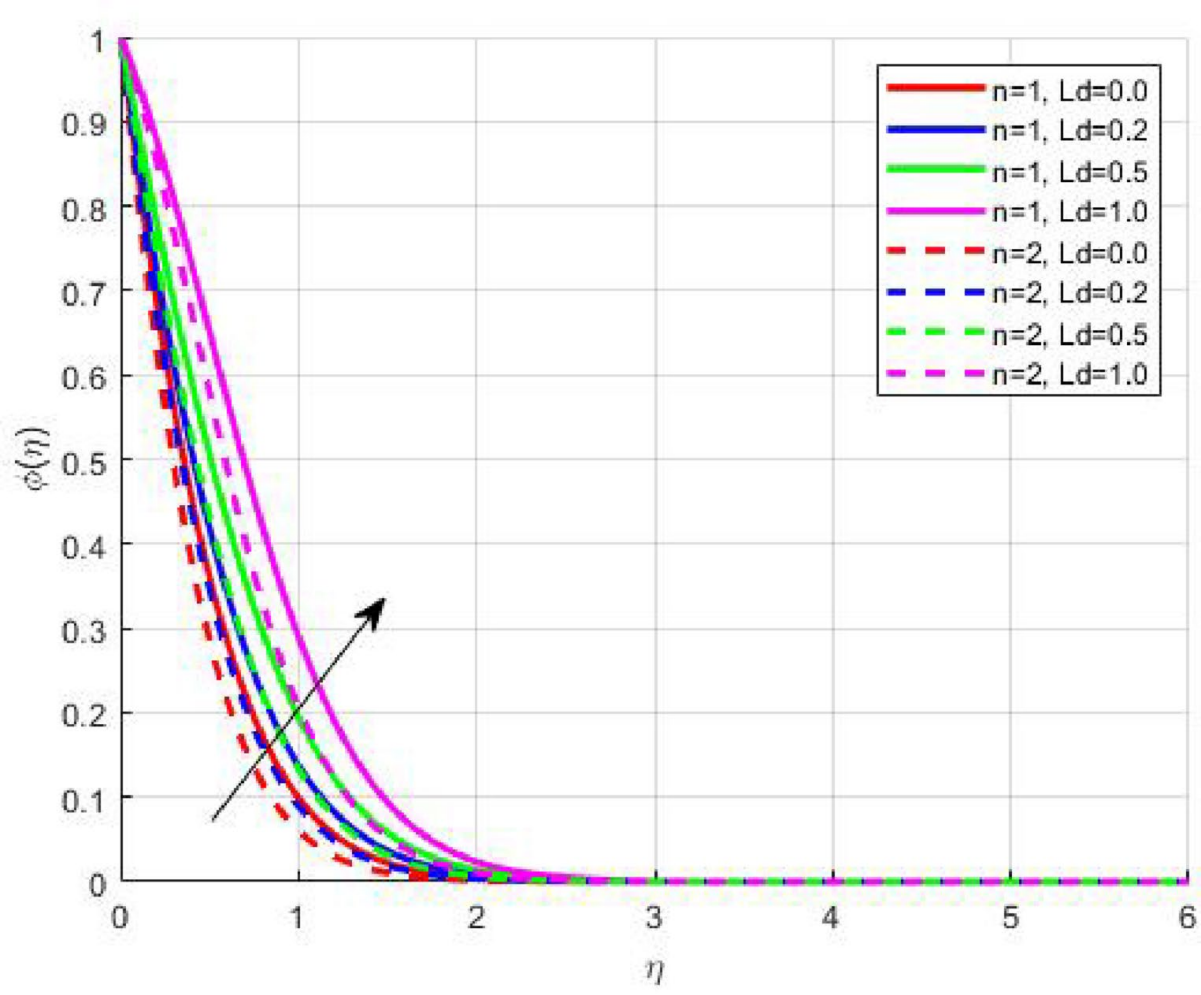
$$\:\phi\:\left(\eta\:\right)$$ on $$\:Ld$$.

**Fig. 17:**
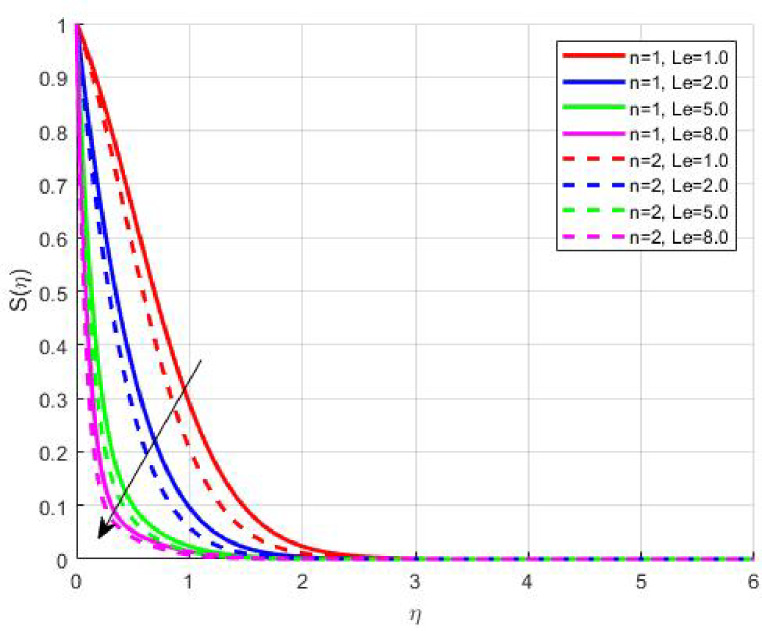
$$\:S\left(\eta\:\right)$$ on $$\:Le$$.

**Fig. 18:**
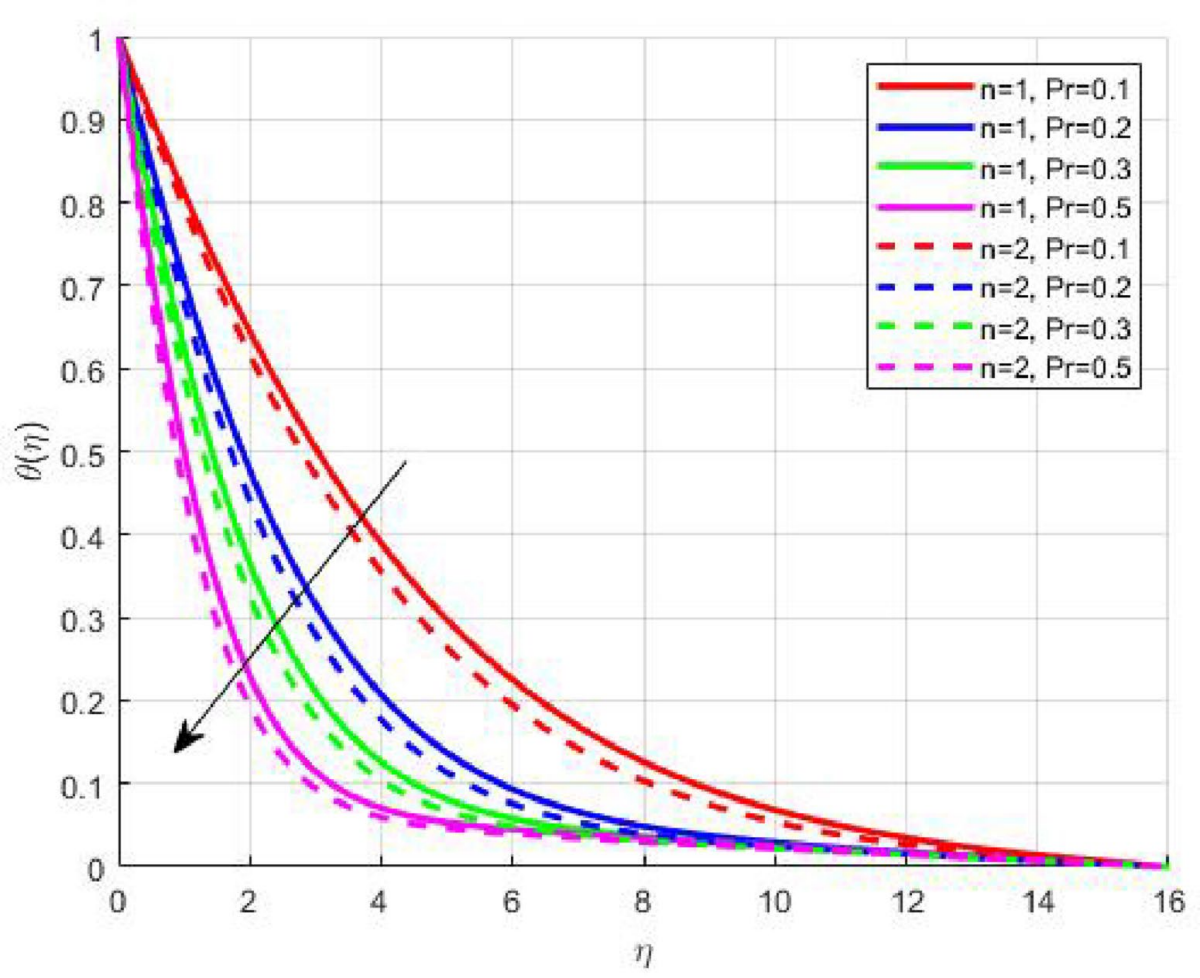
$$\:\theta\:\left(\eta\:\right)$$ on $$\:Pr$$.

**Fig. 19:**
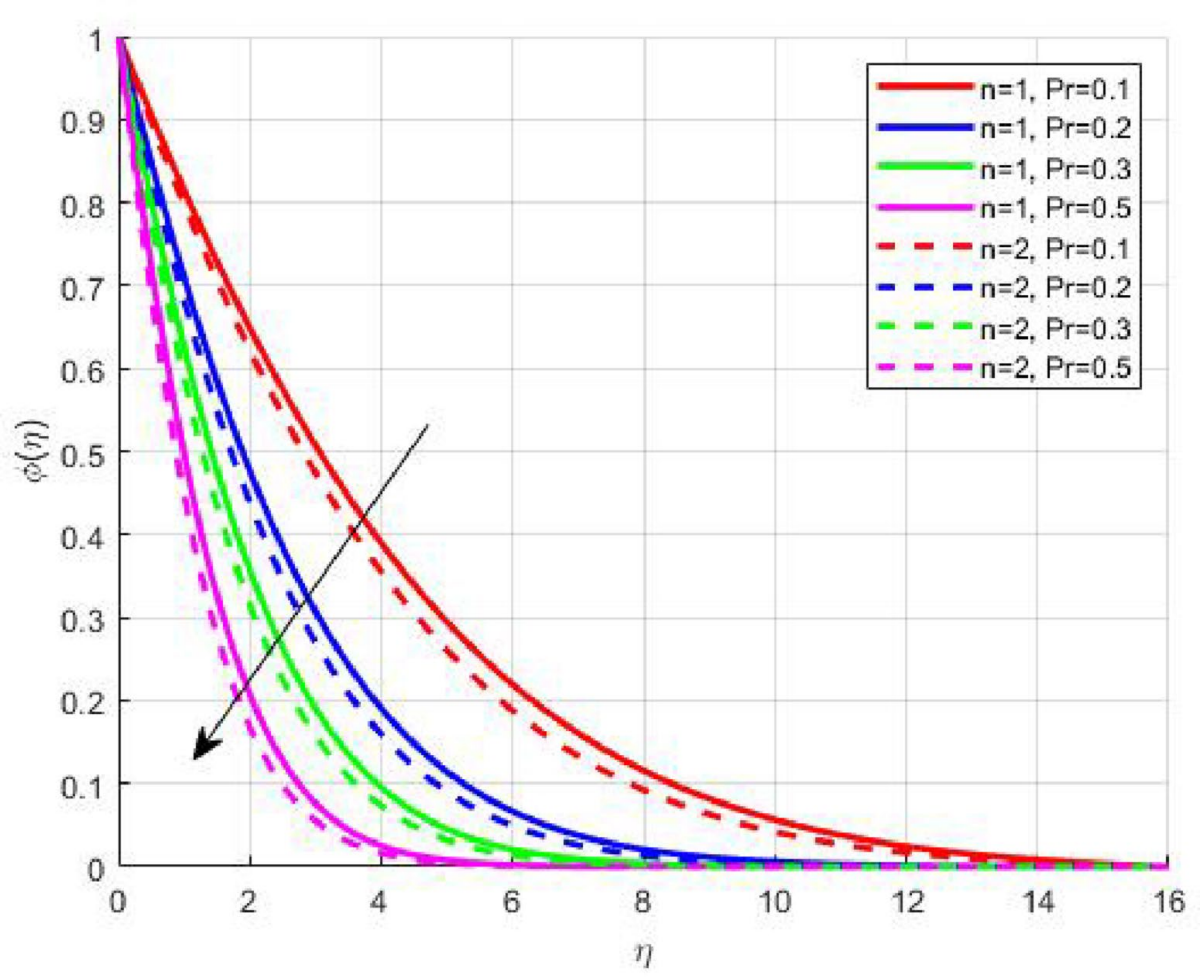
$$\:\phi\:\left(\eta\:\right)$$ on $$\:Pr$$.

**Fig. 20:**
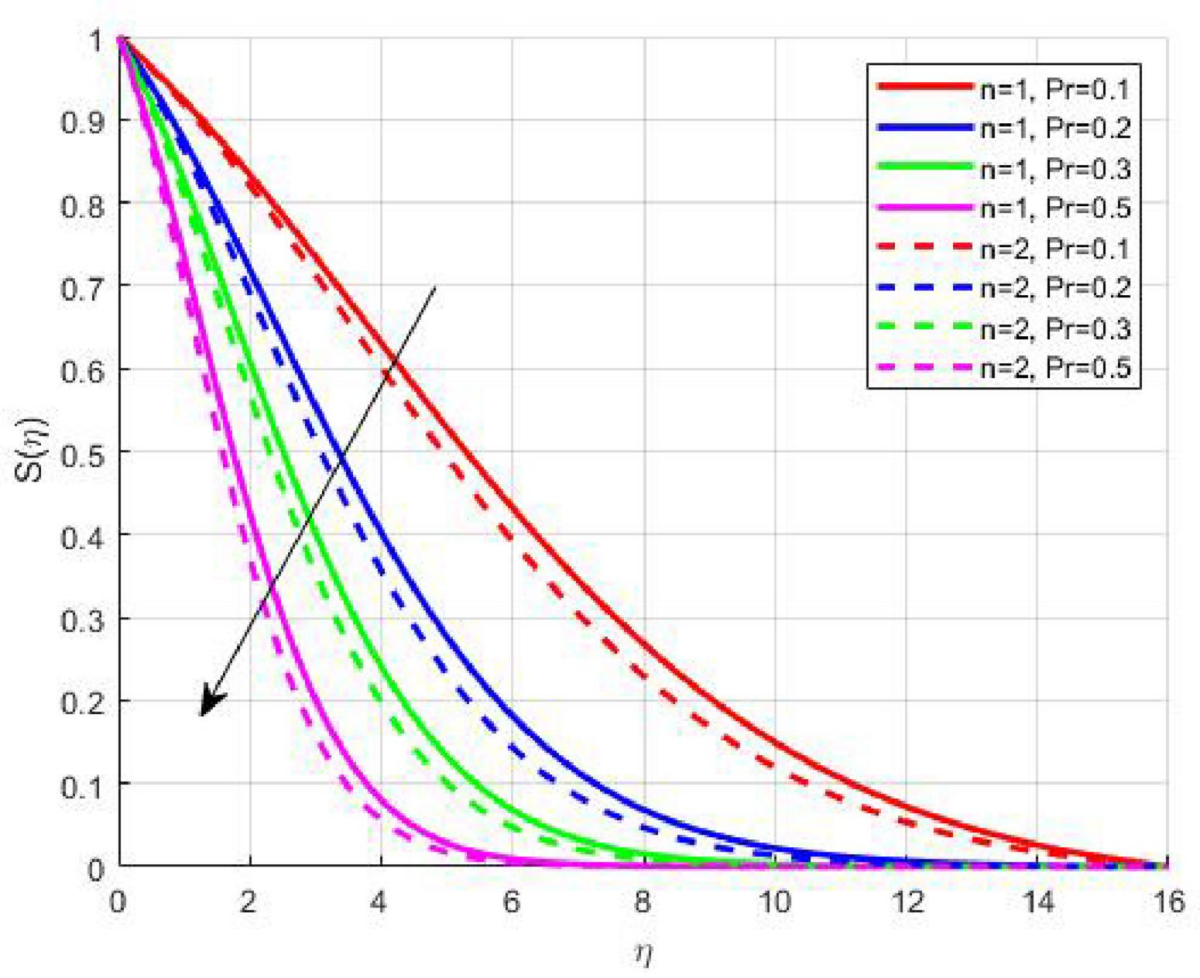
$$\:S\left(\eta\:\right)$$ on $$\:Pr$$.

**Fig. 21:**
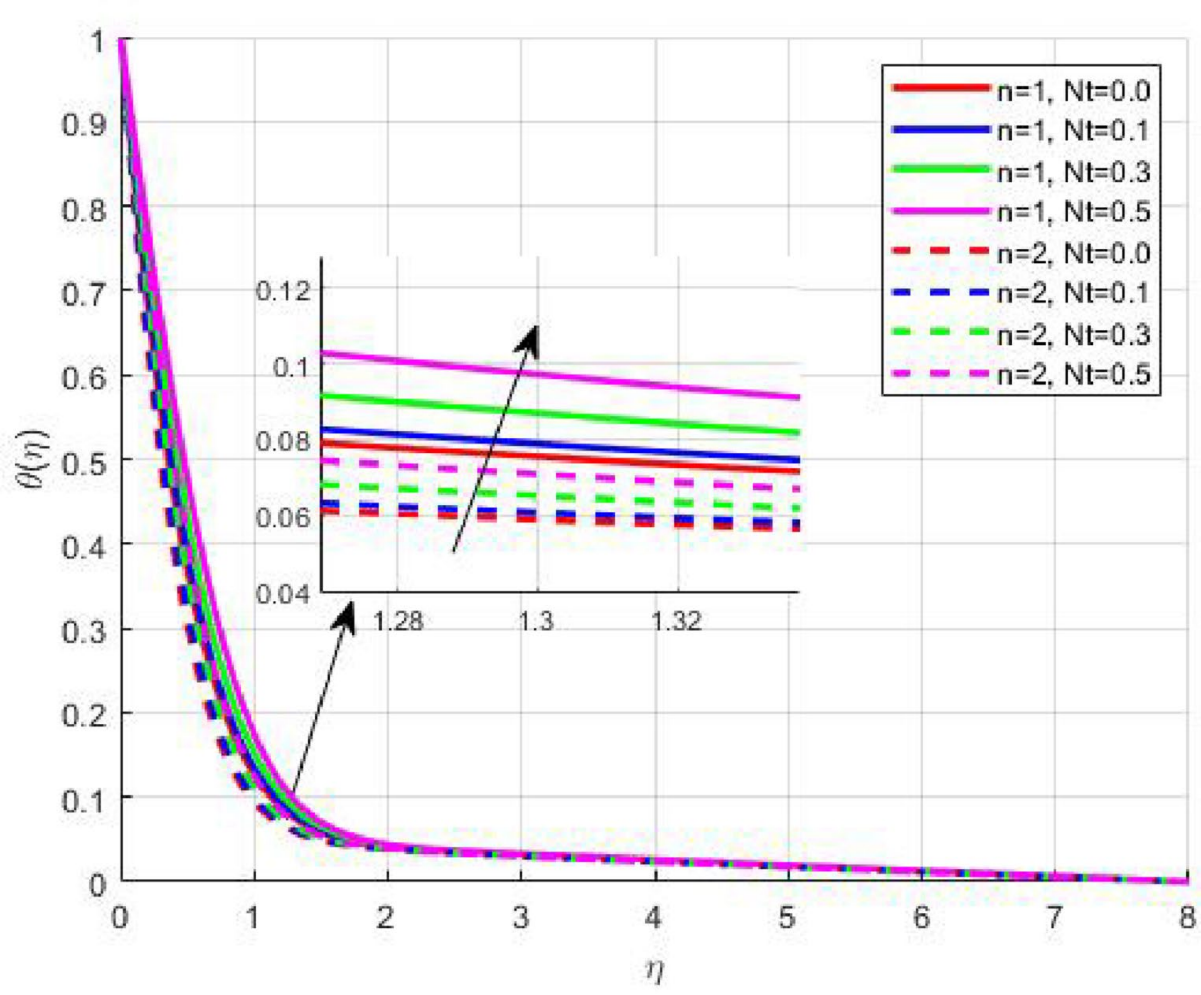
$$\:\theta\:\left(\eta\:\right)$$ on $$\:Nt$$.

**Fig. 22:**
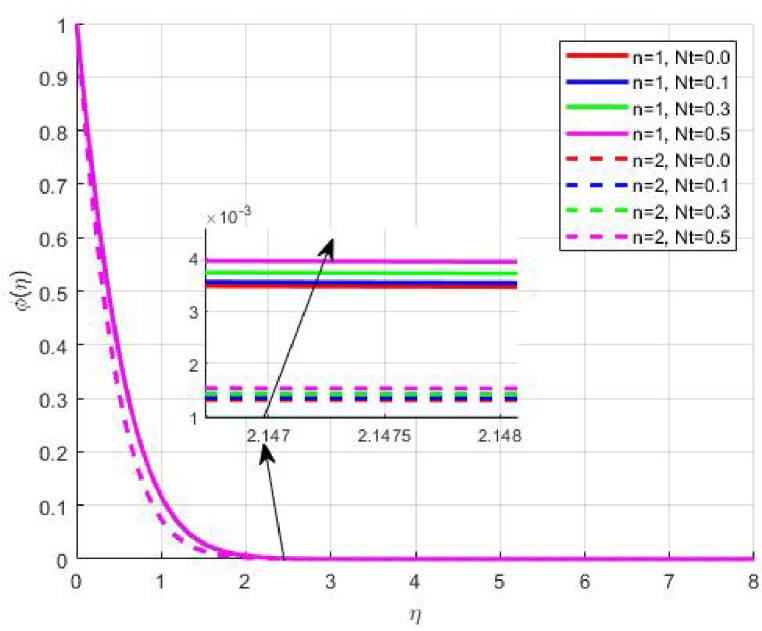
$$\:\phi\:\left(\eta\:\right)$$ on $$\:Nt$$.

**Fig. 23:**
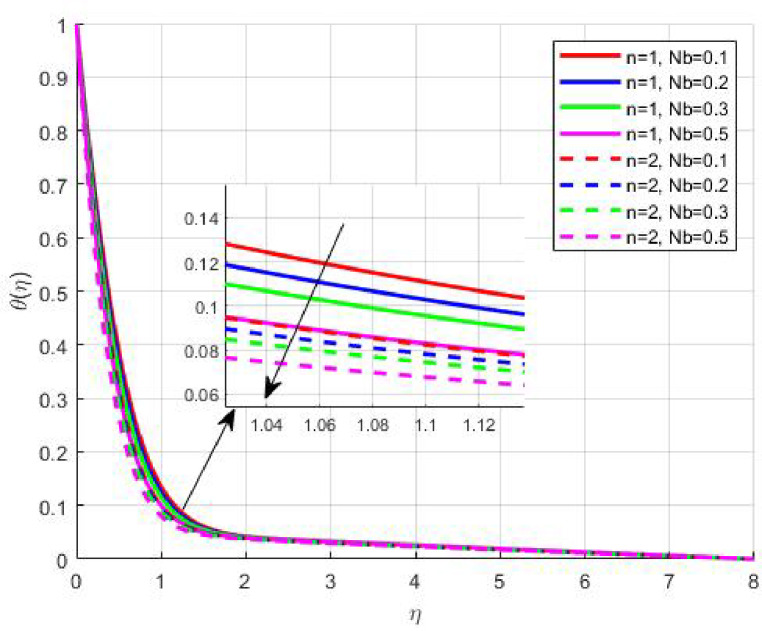
$$\:\theta\:\left(\eta\:\right)$$ on $$\:Nb$$.

**Fig. 24:**
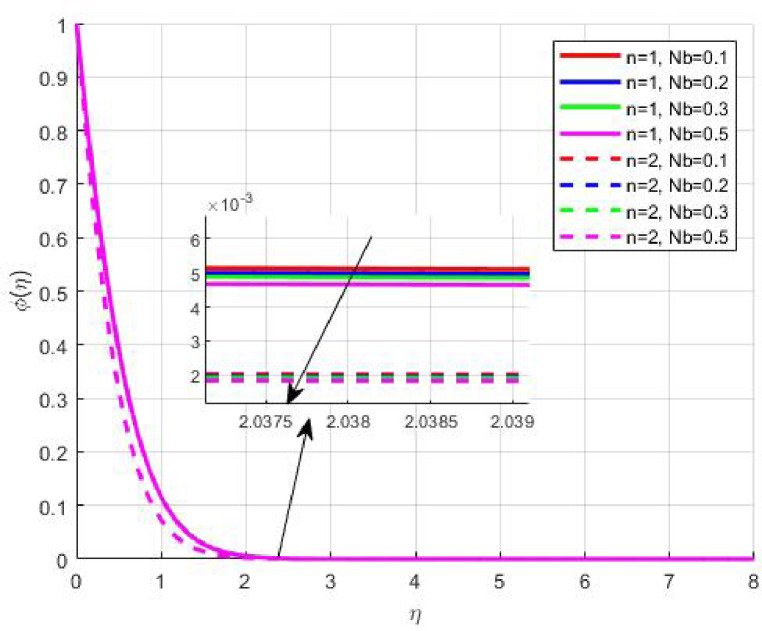
$$\:\phi\:\left(\eta\:\right)$$ on $$\:Nb$$.

**Fig. 25:**
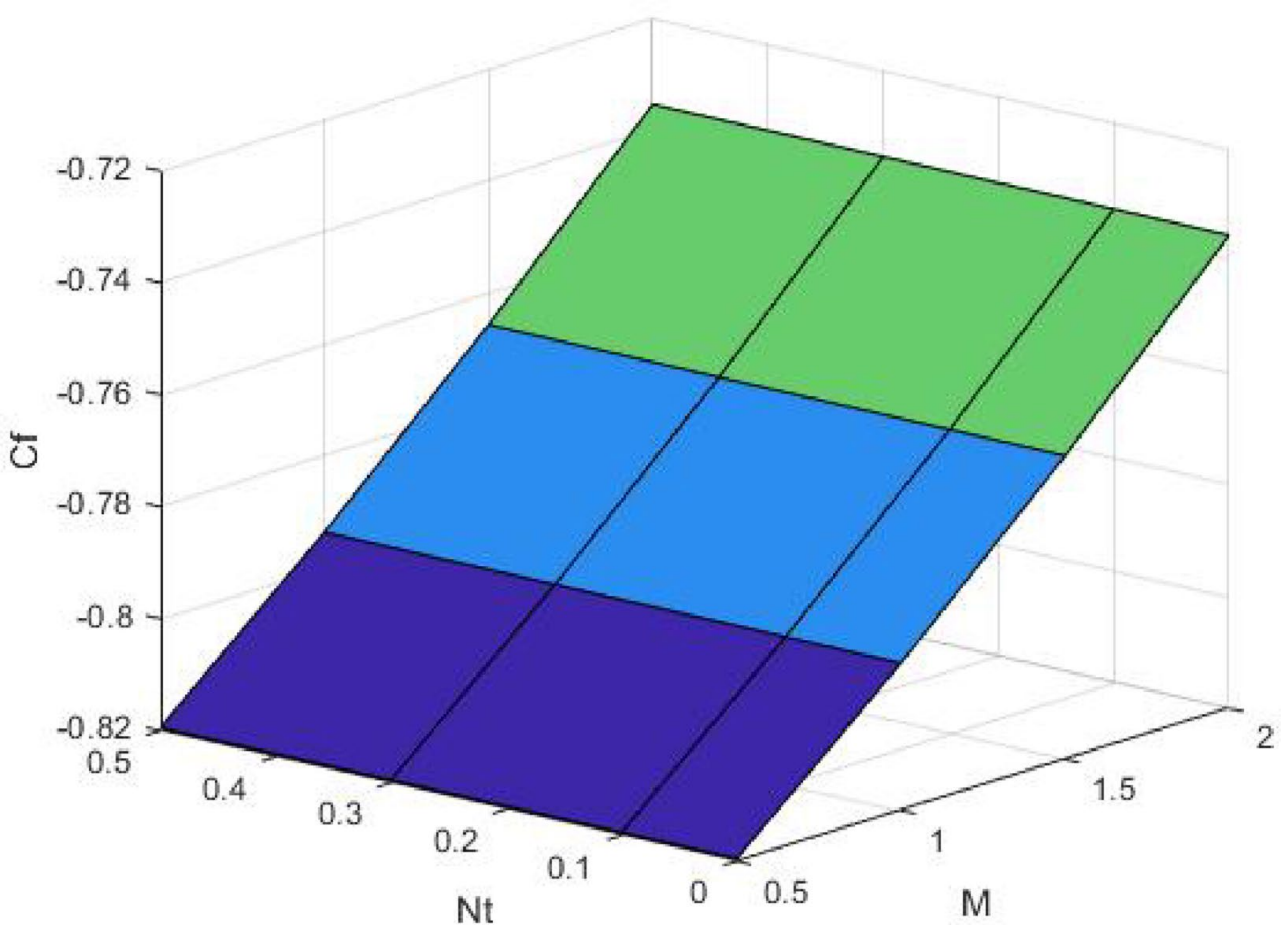
$$\:Cf$$ on $$\:Nt\:$$and M.

**Fig. 26:**
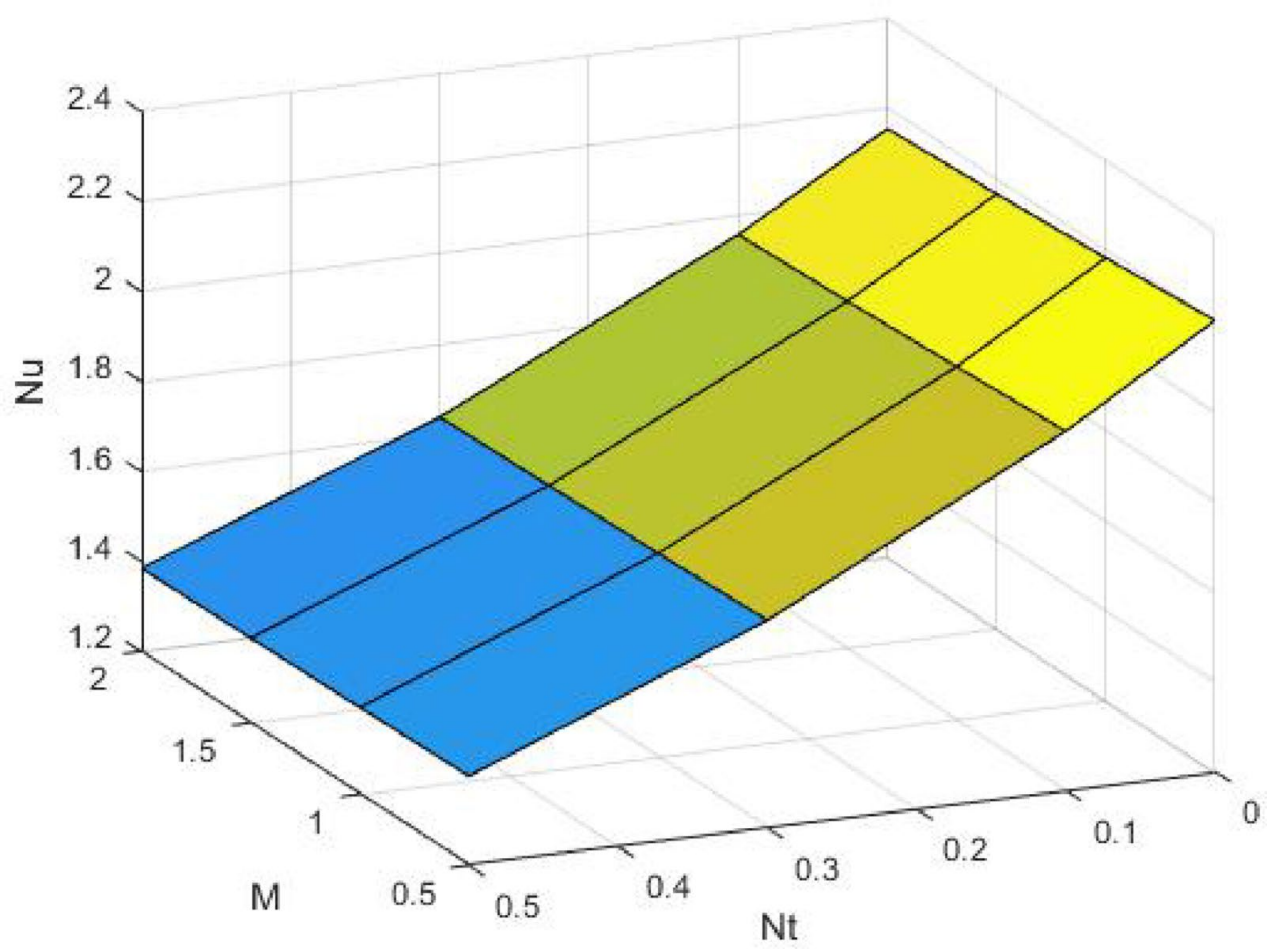
$$\:Nu$$ on $$\:Nt\:$$and M.

**Fig. 27:**
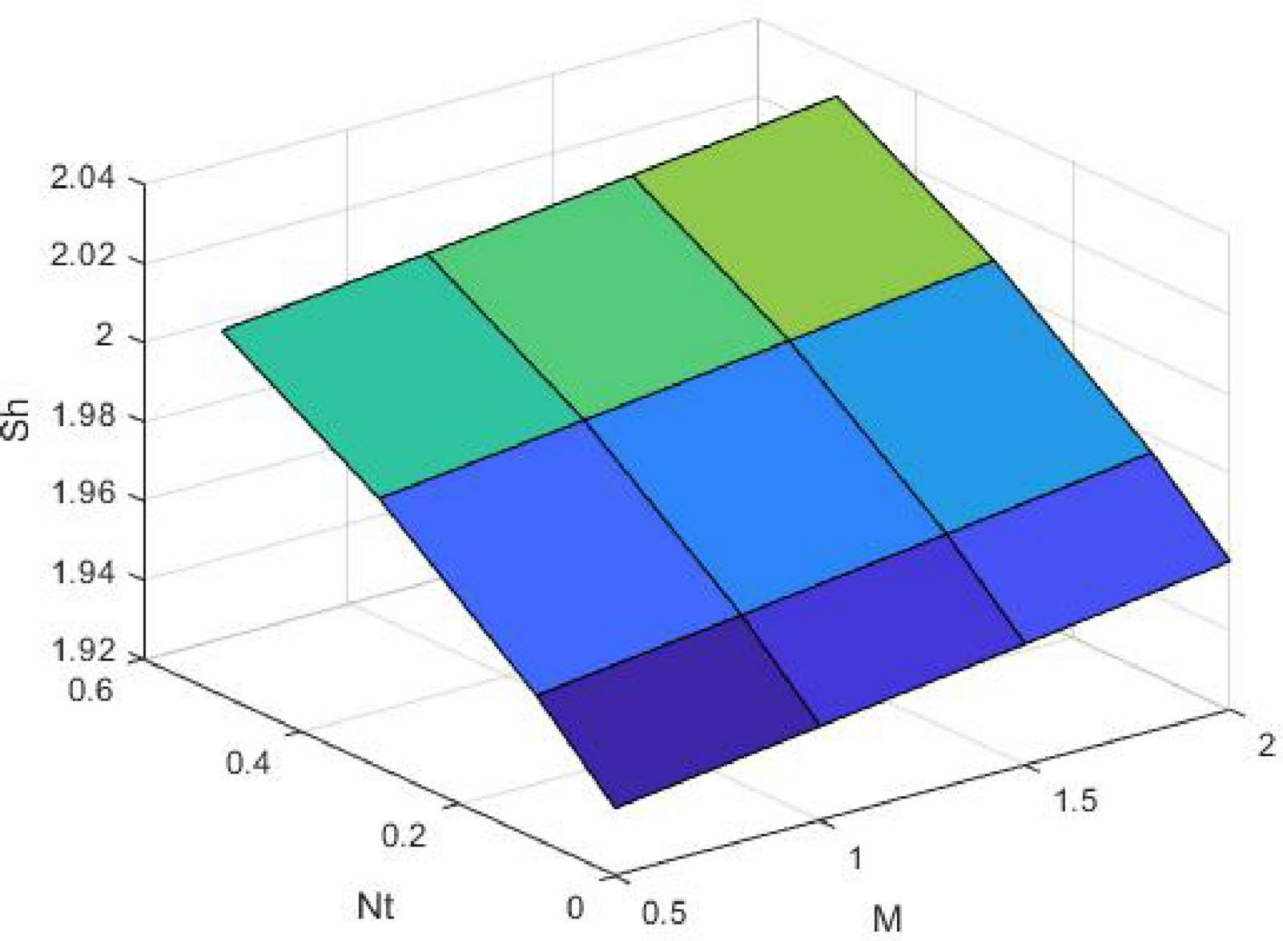
$$\:Sh$$ on $$\:Nt\:$$and M.

**Fig. 28:**
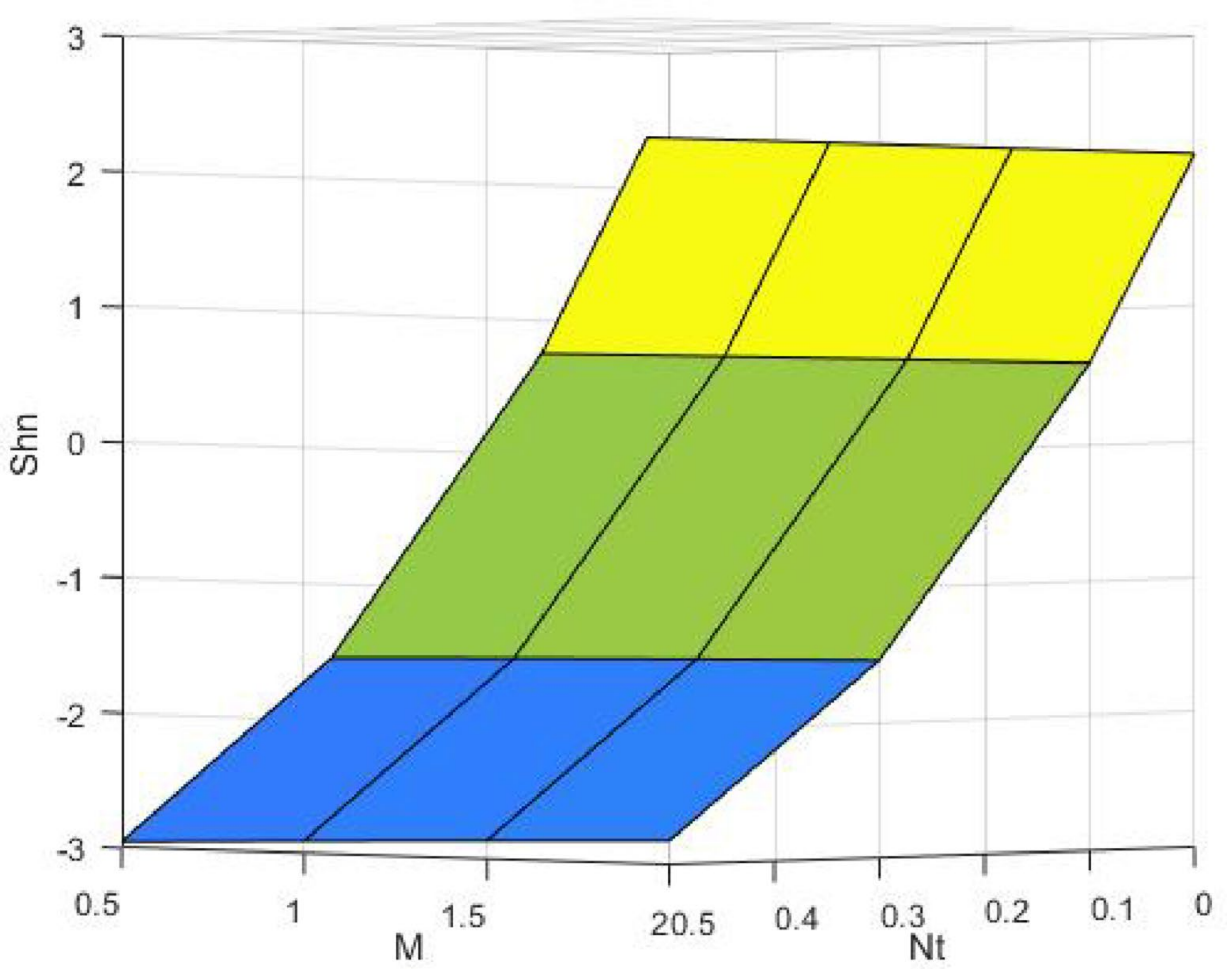
$$\:Shn$$ on $$\:Nt\:$$and M.

Figures 15, 16 and 17 investigate how the Lewis number (Le) governs nanoparticle and solutal concentration fields. Increasing Le—which represents the ratio of thermal to mass diffusivity—results in a marked decrease in concentration. When Le is high, mass diffusivity dominates over thermal diffusion, accelerating species migration away from the wall and producing thinner concentration layers. This implies a more rapid homogenization of nanoparticles and solutes in the fluid bulk. Conversely, at intermediate Le values, a balanced diffusive interaction enhances retention of nanoparticles near the surface, improving local thermal conductivity. Such controlled diffusion behaviour is critical in processes like catalytic coating, nanofluid-based heat exchangers, and drug delivery systems, where precise mass transfer regulation ensures optimal performance. The influence of the Prandtl number (*Pr*) on temperature and concentration fields is depicted in Figs. 18, 19 and 20. An elevation in *Pr* corresponds to reduced thermal diffusivity, leading to lower temperature, nanoparticle, and solutal concentrations throughout the boundary layer. This occurs because a higher *Pr* limits thermal energy propagation, thereby confining heat to the near-wall region and minimizing its diffusion into the fluid bulk. Consequently, both the thermal and solutal boundary layers become thinner. Physically, this behaviour reflects a fluid with high viscosity and poor thermal conduction—characteristics common to oils or polymeric solutions. Such trends are advantageous for applications where localized temperature control or insulation is desired, such as in polymer processing or temperature-sensitive manufacturing. Figures 21 and 22 exhibit the impact of the thermophoretic parameter (Nt) on temperature and nanoparticle concentration. As Nt rises, both temperature and concentration increase significantly. Thermophoresis induces nanoparticle motion from hotter to cooler zones, but higher Nt amplifies the overall particle migration, causing heat and mass redistribution across the boundary layer. This enhances the effective thermal conductivity and particle density near cooler regions. Physically, stronger thermophoretic forces assist in spreading thermal energy uniformly, which is valuable in microscale cooling, solar collectors, and nanoparticle-based heat exchangers where efficient energy transport is essential. The influence of Brownian motion parameter (*Nb*) on temperature and nanoparticle concentration is depicted in Figs. 23 and 24 with increasing *Nb*, both temperature and concentration profiles exhibit higher values due to intensified random particle motion. Enhanced Brownian diffusion promotes microscopic mixing of nanoparticles, elevating heat conduction and producing more uniform energy distribution across the flow field. This phenomenon mitigates localized temperature gradients, improving stability and efficiency in applications such as industrial mixers, advanced lubricants, and thermal storage devices where homogeneous nanoparticle dispersion is critical. Figures 25, 26, 27 and 28 illustrate three-dimensional surface plots showing the interplay between the magnetic field parameter (*M*) and the thermophoretic parameter (*Nt*). The velocity profile declines with higher *M* due to the Lorentz force opposing motion, whereas temperature and concentration fields intensify with *Nt* because of enhanced thermophoretic diffusion. These contrasting effects underscore a competitive mechanism: while magnetization resists flow and limits momentum transfer, thermophoresis enhances energy and mass redistribution. Such an interactive response is central to designing magnetically actuated cooling systems, electro-conductive nanofluid channels, and energy harvesting devices where field-controlled transport is vital for optimizing performance.

**Fig. 29:**
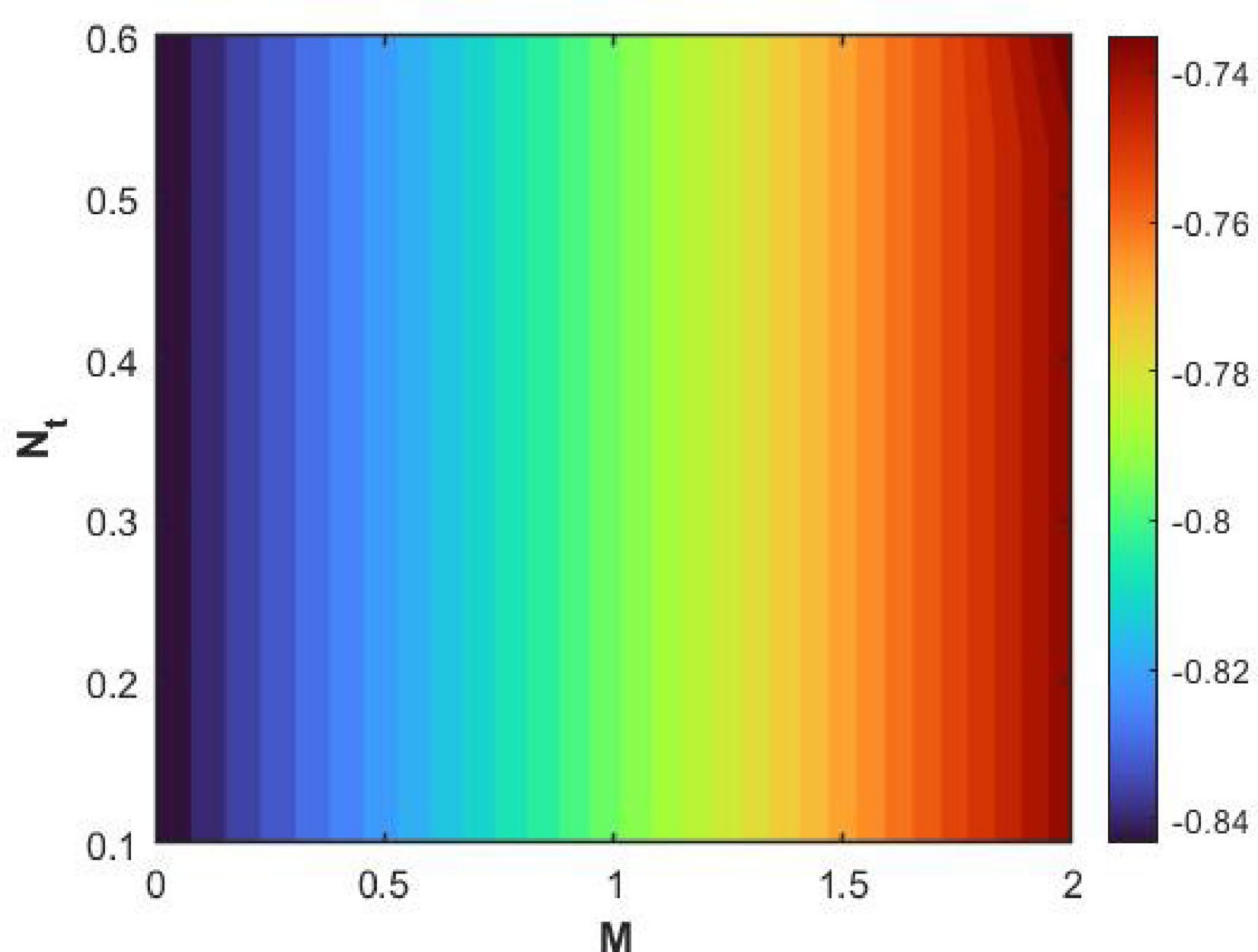
Contour plot of $\:Cf$.

**Fig. 30:**
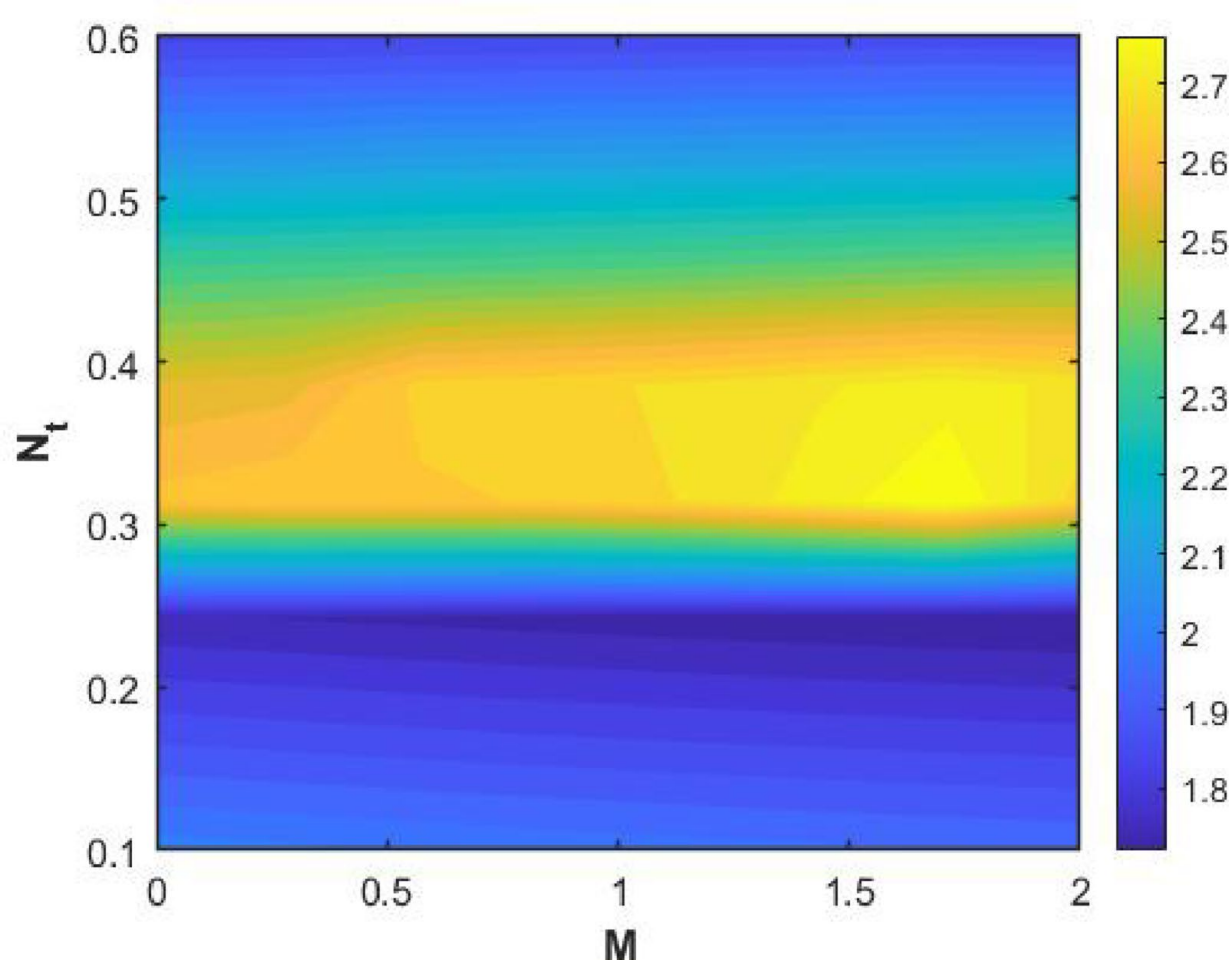
Contour plot of $\:Nu$.

**Fig. 31:**
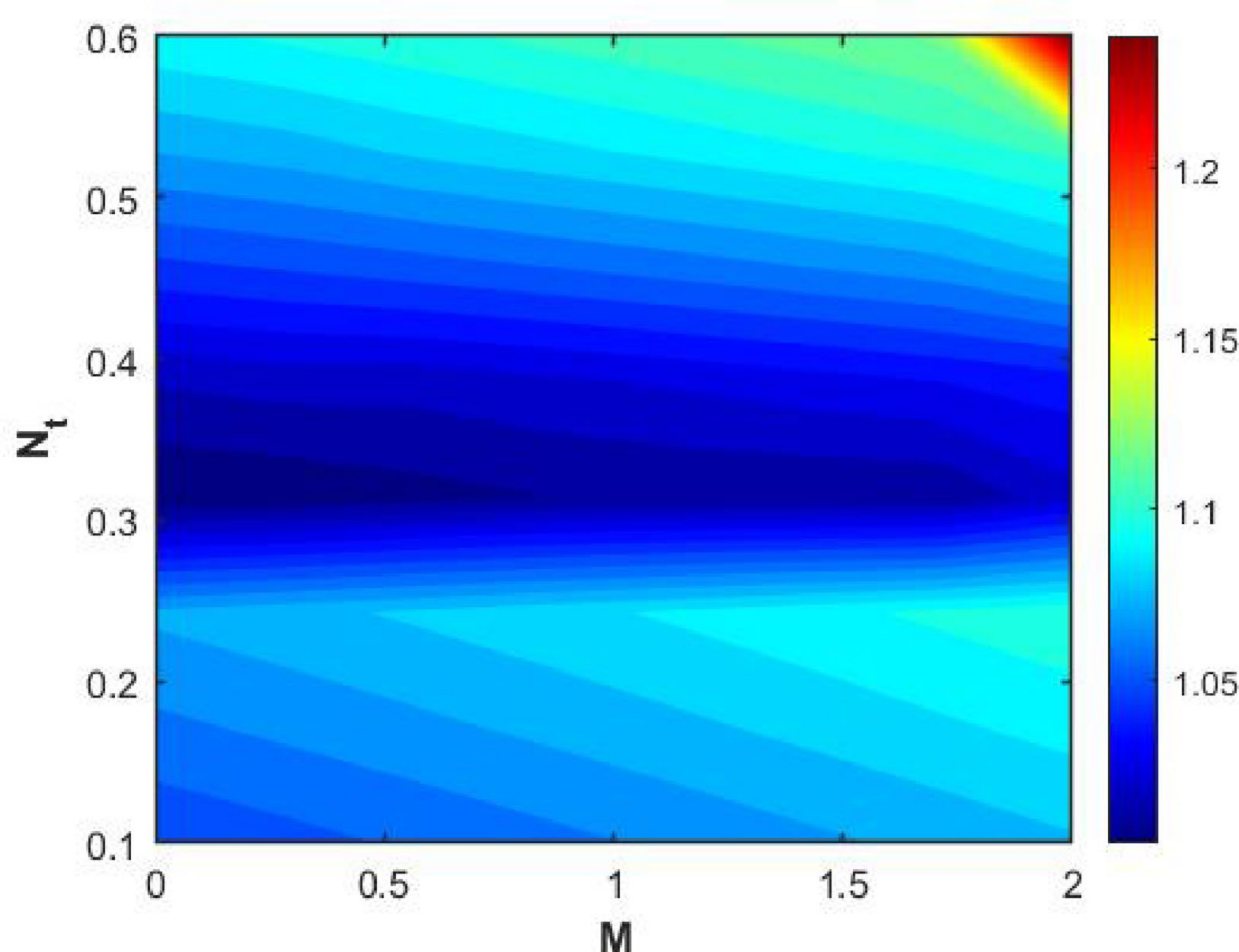
Contour plot of $\:Sh$.

**Fig. 32:**
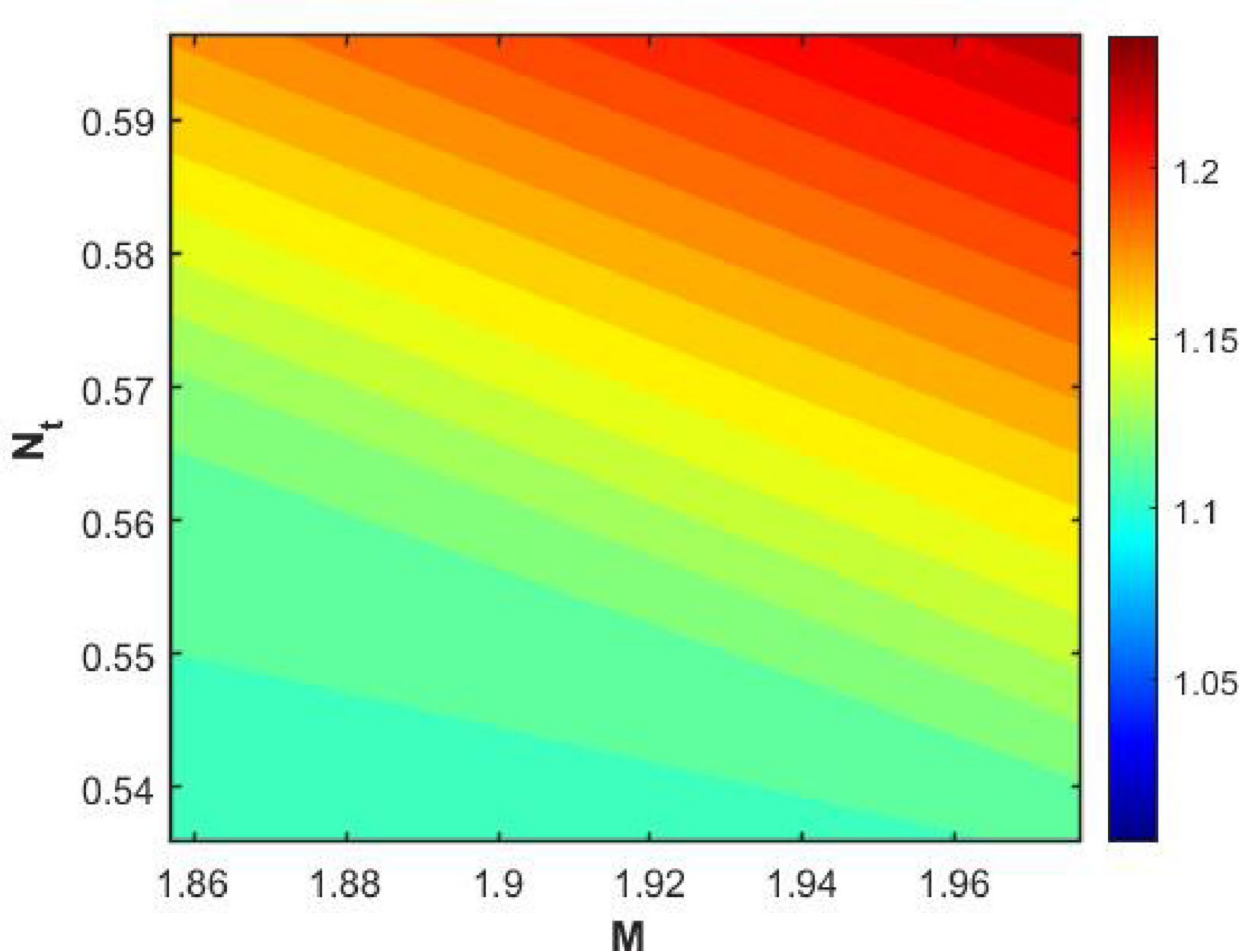
Contour plot of $\:Shn$.

**Fig. 33:**
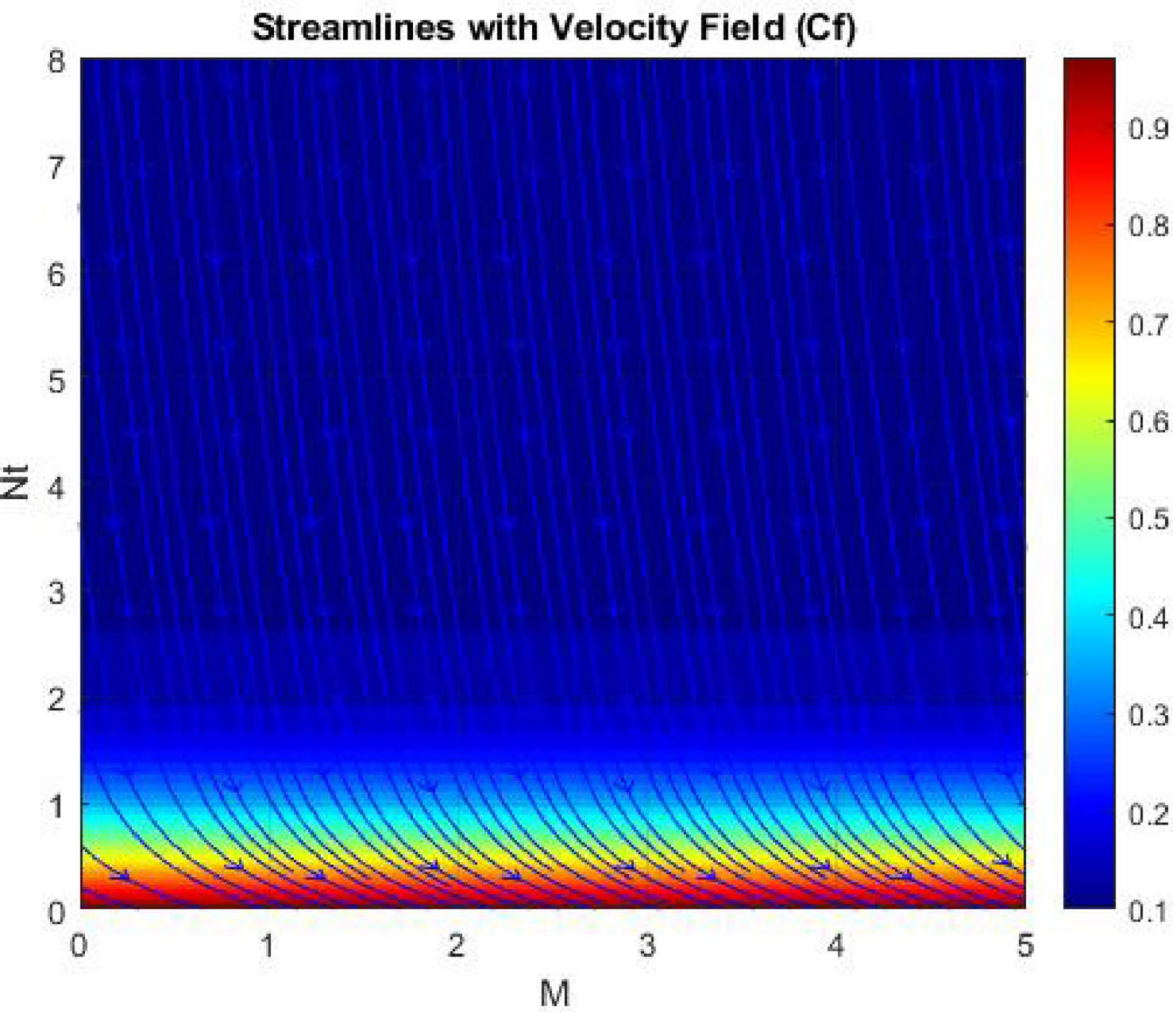
Stream lines of $\:Cf$.

**Fig. 34:**
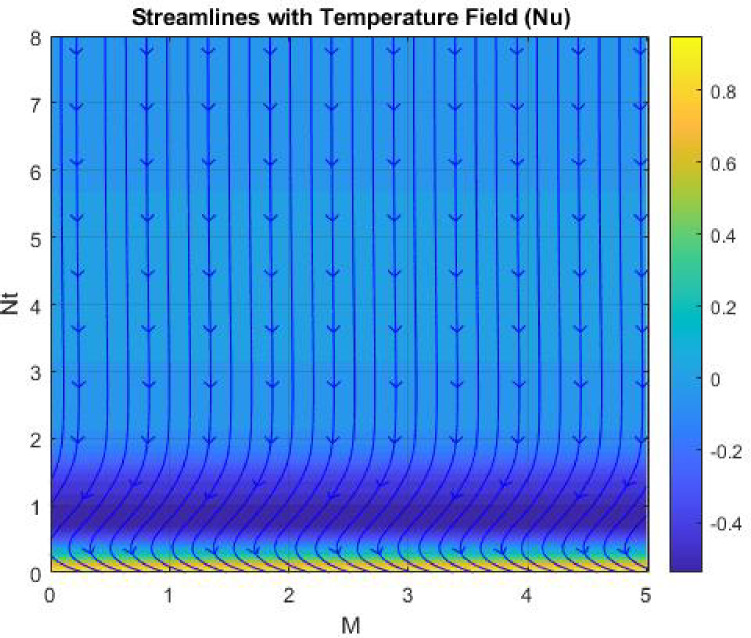
Stream lines of $\:Nu$.

**Fig. 35:**
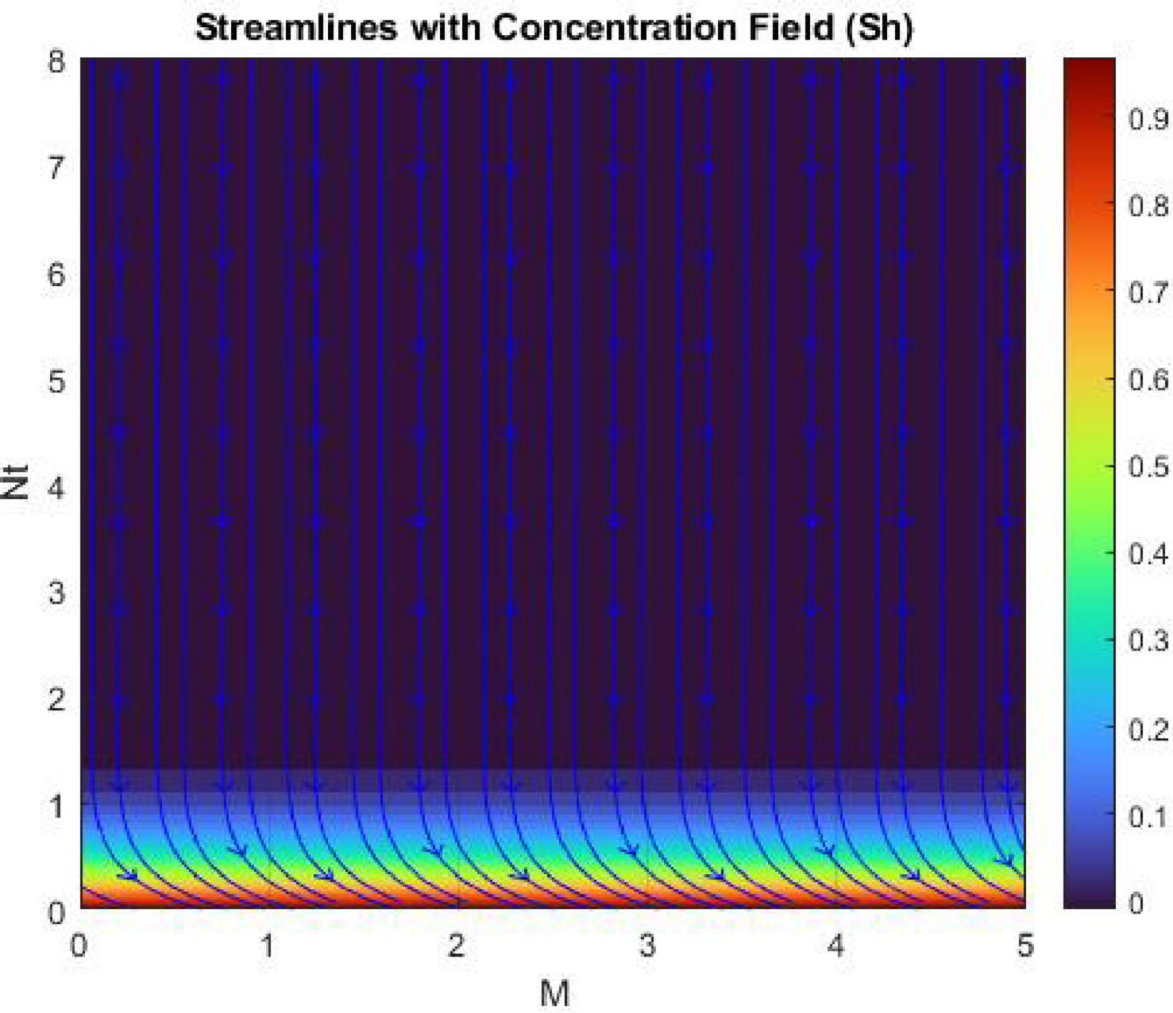
Stream lines of $\:Sh$.

**Fig. 36:**
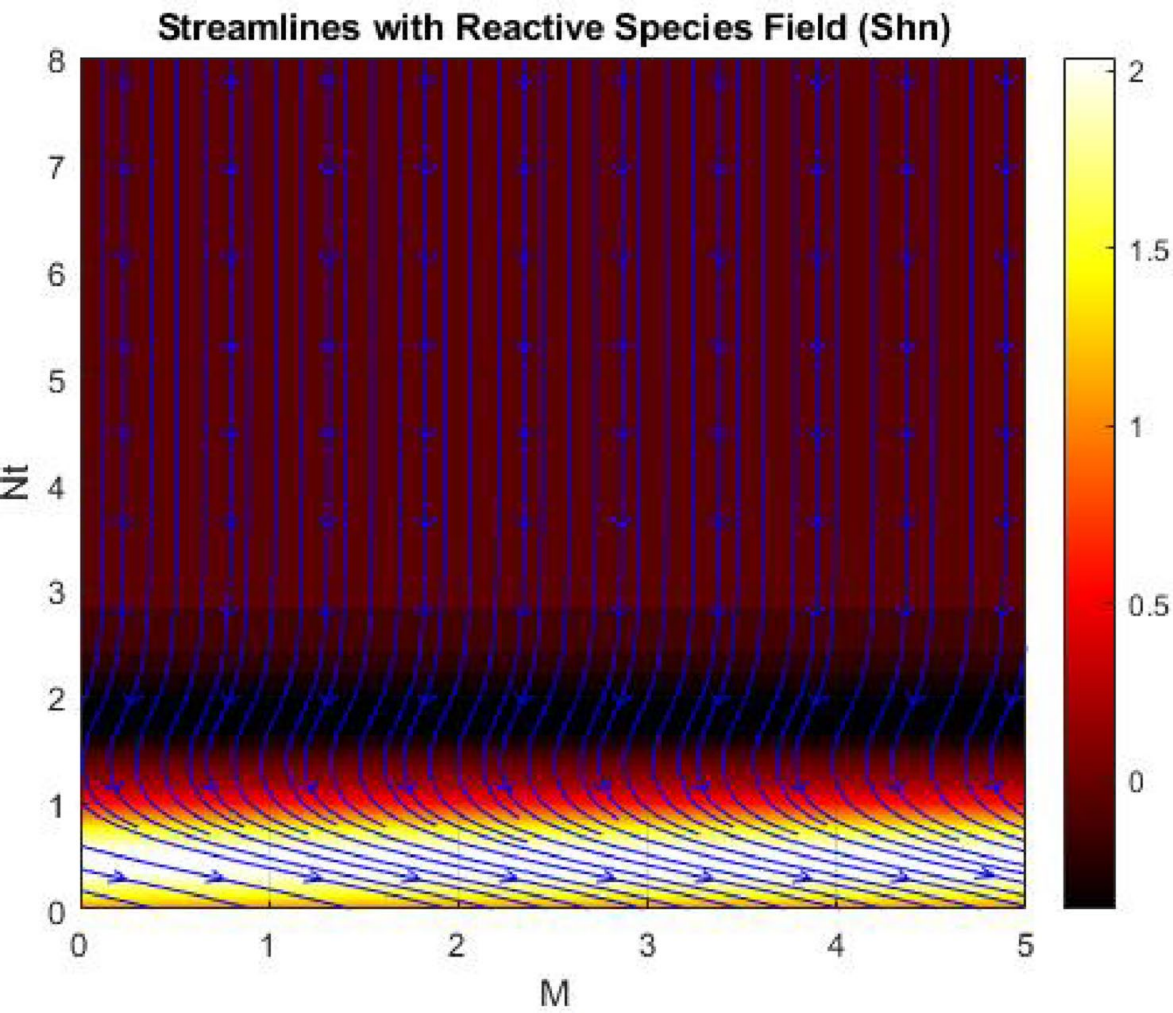
Stream lines of $\:Shn$.

Figures 29, 30, 31 and 32 provide contour representations of how magnetic and thermophoretic forces modulate fluid flow, temperature, and species transport characteristics. The contour distributions delineate gradients of velocity and concentration, revealing how the magnetic field suppresses motion while thermophoresis redistributes nanoparticles across the domain. These contours highlight the intricate coupling between electromagnetic damping and nanoparticle migration, offering valuable insights for coating technologies, reactive fluid systems, and nanofluid-based energy devices. By controlling the interplay of M and Nt, engineers can tailor heat and mass transfer patterns to improve system uniformity and efficiency. Figures 33, 34, 35 and 36 display streamline plots that elucidate the flow topology under simultaneous magnetic and thermophoretic influences. As the magnetic parameter increases, the streamlines become denser and closer to the surface, signifying flow retardation and reduced recirculation. Conversely, higher thermophoretic strength broadens the streamline distribution, indicating stronger thermal migration and enhanced mixing. These opposing trends reveal a dual-control mechanism, where magnetic suppression and thermophoretic activation jointly dictate energy and momentum distribution. Such understanding is instrumental in designing microfluidic devices, magnetic-field-assisted reactors, and compact heat exchangers that rely on precise flow manipulation for optimal thermal and mass transport outcomes. Overall, the results confirm that suction and magnetic field intensify resistance to motion, while thermophoresis and Brownian motion promote heat and mass dispersion. The Lewis and Prandtl numbers further regulate diffusivity and viscosity-driven behaviours, defining the relative dominance of conduction versus convection. The combined parametric influence demonstrates a tenable transport mechanism that can be engineered for enhanced thermal control, energy efficiency, and flow regulation in nanofluid-based technologies.

**Fig. 37:**
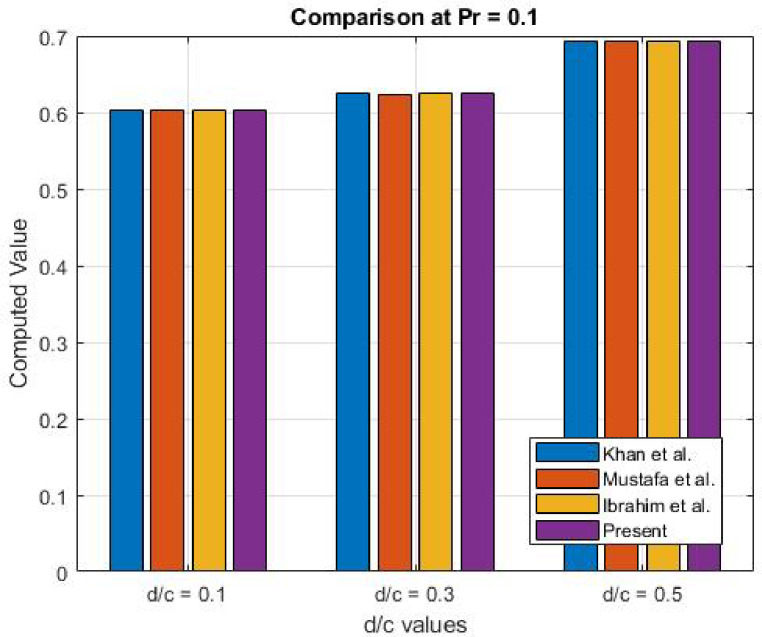
Comparison at Pr (0.1).

**Fig. 38:**
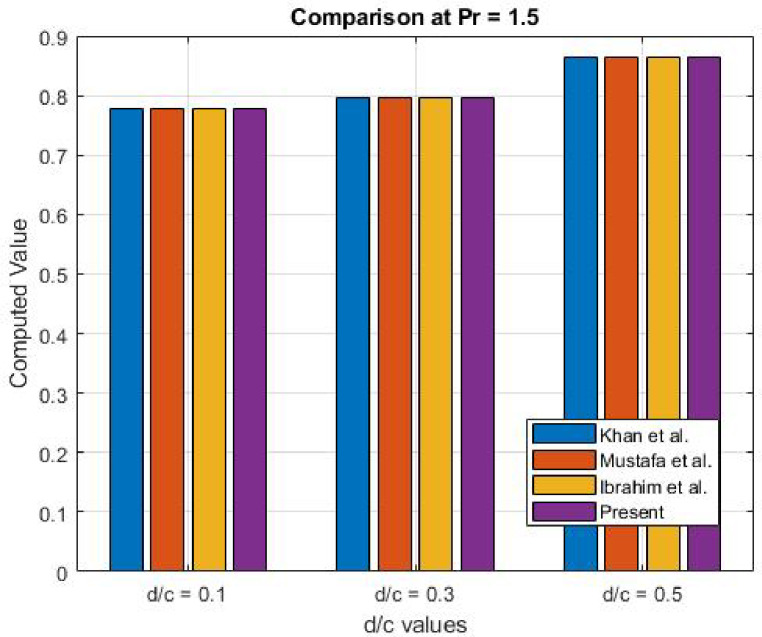
Comparison at Pr (1.5).

Despite the comprehensive analysis presented, this study has certain inherent limitations that define its current scope. The investigation is confined to a steady, laminar, and two-dimensional nanofluid flow configuration, thereby neglecting unsteady, turbulent, or three-dimensional influences that may occur in practical systems. Furthermore, the thermophysical properties of the fluid are treated as constant, and several complex real-world factors—such as spatially varying magnetic fields, surface irregularities, chemical reactions, and non-uniform viscous dissipation effects—are not considered in the present formulation. Additionally, the outcomes are derived solely through numerical simulation without experimental validation, which may constrain the direct applicability of the results to industrial or experimental environments. These acknowledged simplifications, however, serve to establish a foundational understanding upon which more advanced models can be built in future investigations.

These visualizations help understand how fluid movement changes in response to varying parameters like suction and magnetic fields, which directly affect heat and mass transfer. In practical applications like micro-scale cooling systems or nanofluid-based heat exchangers, these flow patterns can guide the optimization of system design to ensure uniform distribution of heat and nanoparticles. Together, the results from these figures provide a comprehensive understanding of how suction, magnetic fields, and thermophoretic effects influence nanofluid behaviour. This knowledge is crucial for a wide range of engineering applications where precise control of heat, mass transfer, and fluid flow is essential for achieving optimal system performance. The computed outcomes in Table 1 have broad significance across numerous scientific and engineering domains dealing with nanofluid-based heat and mass transfer processes. The skin friction coefficient denotes the surface shear, which plays a crucial role in applications such as coating flows and polymer sheet stretching. The Nusselt number, which quantifies thermal convection, aids in improving the design of effective heating and cooling technologies, including electronic device cooling and thermal regulation systems. The Sherwood numbers measure solute and nanoparticle diffusion, which are vital in fields like chemical processing and medical technologies, particularly for enhancing nanoparticle-driven drug delivery. Adjustments in Brownian motion (Nb) and thermophoresis (Nt) parameters highlight the influence of particle-scale transport on temperature distribution, offering valuable information for tailoring nanostructured thermal surfaces. Furthermore, the magnetic field parameter (M) is relevant to magnetohydrodynamic (MHD) systems, such as those used in microfluidic controls, advanced manufacturing, and energy equipment. Collectively, these findings facilitate precise modelling and emphasize the importance of parameter sensitivity in optimizing nanofluid flow systems under varying physical conditions.

**Table 1:** Numerical values of physical parameters.

A	M	Nb	Nt	Nd	Ld	Cf	Nu	Sh	Shn
**0.5**	1.0	0.1	0.1	0.1	0.1	0.26437	0.25889	0.69057	0.35931
**0.6**	1.0	0.1	0.1	0.1	0.1	0.22517	0.21552	0.69401	0.22801
**0.7**	**1.0**	0.1	0.1	0.1	0.1	0.29452	0.17475	0.69722	0.11814
0.7	**1.5**	0.1	0.1	0.1	0.1	0.29541	0.11028	0.70226	0.17073
0.7	**2.0**	**0.1**	0.1	0.1	0.1	0.29557	0.04454	0.70742	0.22446
0.7	2.0	**0.2**	0.1	0.1	0.1	0.29557	0.04885	0.68026	0.22132
0.7	2.0	**0.3**	**0.1**	0.1	0.1	0.29557	0.05327	0.65247	0.21808
0.7	2.0	0.3	**0.2**	0.1	0.1	0.29545	0.04273	0.65528	0.30400
0.7	2.0	0.3	**0.3**	**0.1**	0.1	0.29535	0.03340	0.65780	0.38595
0.7	2.0	0.3	0.3	**0.2**	0.1	0.29525	0.01417	0.74703	0.40245
0.7	2.0	0.3	0.3	**0.3**	**0.1**	0.29524	0.00481	0.83321	0.41888

**Table 2:** Comparison of numerical results $\:-{\theta\:}^{{\prime\:}}\left(0\right)$.

Pr	d/c	^23^	^24^	^25^	Present
0.1	0.1	0.6022	0.6021	0.6022	0.602547
0.1	0.3	0.6255	0.6244	0.6255	0.625496
0.1	0.5	0.6924	0.6924	0.6924	0.692345
1.5	0.1	0.7768	0.7768	0.7768	0.776901
1.5	0.3	0.7971	0.7971	0.7971	0.797084
1.5	0.5	0.8648	0.8647	0.8648	0.864785

### Results validation

Table 2 showcases a comparison of the surface temperature obtained from the numerical results of Khan et al.^23^, Mustafa et al.^24^, Ibrahim et al.^25^, and the present study. The temperature values from the current investigation are in close agreement with those reported in earlier studies, which affirms the accuracy and reliability of the employed numerical technique. This comparison not only serves to validate the results but also highlights the consistency across different research efforts, reinforcing the robustness of the present method for analysing nanofluid flow and heat transfer dynamics. Figures 37 and 38 offer a visual comparison of the outcomes in the form of 3D graphs. Table 3 illustrates the error comparison between the present numerical findings for the surface temperature gradient $\:(-\theta\:{\prime\:}(0\left)\right)$ and those reported in references^23,24^, and^25^, across various Prandtl numbers (Pr) and velocity ratios (d/c). The errors are minimal, mostly ranging between 10⁻⁴ and 10⁻⁵, demonstrating a very close match with existing literature. The largest discrepancy observed is 0.001096 for Pr = 0.1 and d/c = 0.3 relative to^24^. These results confirm the accuracy and consistency of the current computational method, validating its reliability and alignment with previously established studies.

**Table 3:** Error analysis.

Pr	d/c	Error w.*r*.t^23^	Error w.*r*.t^24^	Error w.*r*.t^25^
0.1	0.1	0.000347	0.000447	0.000347
0.1	0.3	0.000004	0.001096	0.000004
0.1	0.5	0.000055	0.000055	0.000055
1.5	0.1	0.000101	0.000101	0.000101
1.5	0.3	0.000016	0.000016	0.000016
1.5	0.5	0.000015	0.000085	0.000015

## Conclusion

The present study delivers comprehensive insights into the thermos hydrodynamic characteristics of Sisko nanofluid flow with internal heat generation over a stretching sheet situated within a porous medium, incorporating the coupled influences of thermophoresis and Brownian motion. The major findings can be concisely outlined as follows:

- Thermophoretic influence: The thermophoretic force propels nanoparticles from regions of higher to lower temperature, leading to their enhanced accumulation near the surface and thereby augmenting both heat and mass transport rates.
- Brownian motion effect: Brownian agitation facilitates random particle dispersion throughout the fluid, resulting in a more uniform nanoparticle distribution; however, it induces a marginal decline in wall temperature due to elevated molecular energy exchange.
- Suction impact: The application of suction significantly diminishes the velocity, temperature, and nanoparticle concentration fields. Negative suction exhibits a stronger influence, effectively controlling boundary layer thickness and moderating the overall flow behaviour.
- Rheological variation: Alterations in the power-law index notably reshape the flow and thermal profiles, underscoring the pivotal role of non-Newtonian rheology in dictating momentum transfer and diffusion mechanisms.
- Porous medium effect: The resistance imposed by the porous structure contributes to reduced velocity and temperature magnitudes, demonstrating its utility in applications requiring fine-tuned heat dissipation and flow regulation.

In summary, the outcomes highlight the intricate coupling between thermophoretic diffusion, Brownian motion, suction control, and Sisko fluid rheology in shaping nanofluid transport dynamics. These insights furnish a robust theoretical framework for the advancement of thermal engineering systems—including heat exchangers, microchannel cooling configurations, and energy-efficient fluid devices—where precise management of heat and mass transfer is of paramount importance.

## Data Availability

All data analyzed during this study are included and/or properly cited in this article.

## List of symbols

$u,v$: Velocity components in $x$ and $y$ directions ($m/s$)
$T$: Temperature of the fluid ($K$)
$$C$$: Nanoparticle concentration ($$kg/m^3$$)
$\varphi$: Additional nanoparticle concentration due to secondary species ($kg/m^3$)
${\rho }_{f}$: Density of base fluid ($kg/m^3$)
$$\mu$$: Dynamic viscosity ($$Pa\cdot s$$)
$$\sigma$$: Electrical conductivity ($$S/m$$)
${B}_{0}$: Magnetic field strength (*T* (Tesla))
$${\alpha }_{m}$$: Thermal diffusivity ($$m^2/s$$)
$Cp$: Specific heat at constant pressure ($J/(kg\cdot K)$)
${D}_{B}$: Brownian diffusion coefficient ($m^2/s$)
${D}_{T}$: Thermophoretic diffusion coefficient ($kg\cdot m/(s^3\cdot K)$)
$${D}_{S}$$: Solutal diffusivity ($$m^2/s$$)
${D}_{TC}$: Thermal-diffusion cross coefficient for concentration ($m^2/s$)
${D}_{CT}$: Dufour-type cross diffusion coefficient ($m^2/s$)
${U}_{\infty }$: Free-stream velocity ($m/s$)
${u}_{w}$: Surface stretching velocity ($m/s$)
${T}_{w},{T}_{\infty }$: Wall and ambient temperatures (*k*)
${C}_{w},{C}_{\infty }$: Wall and ambient concentrations ($kg/m^3$)
$\varphi_{w},\varphi_{\infty }$: Wall and ambient concentrations of secondary species ($kg/m^3$)
$f(\eta )$: Dimensionless stream function (–)
$$\theta (\eta )$$: Dimensionless temperature (–)
$S(\eta )$: Dimensionless concentration of solute species (–)
$\phi (\eta )$: Dimensionless concentration of secondary species (–)
$$\eta$$: Similarity variable (–)
$n$: Power-law index (Sisko fluid parameter) (–)
$A$: Material parameter ratio (–)
$M$: Magnetic field parameter (–)
$$Pr$$: Prandtl number (–)
$Nb$: Brownian motion parameter (–)
$$Nt$$: Thermophoresis parameter (–)
$$Le$$: Lewis number (–)
$Lb$: Modified Lewis number for secondary species (–)
$Nd$: Dufour-like parameter (–)
$Ld$: Cross-diffusion parameter (–)
$$Ec$$: Eckert number (–)
${Re}_{a},{Re}_{b}$: Reynolds numbers based on different velocity scales (–)
$Cfx$: Local skin friction coefficient (–)
$Nux$: Local Nusselt number (–)
$Shx$: Local Sherwood number for solute species (–)
$Shxn$: Local Sherwood number for secondary species (–)
$\tau$: Ratio of nanoparticle heat capacity to base fluid (–)
$a,b$: Sisko model parameters ($Pa\cdot s^n, kg/(m\cdot s^2)$)
$c$: Stretching rate constant ($1/s$)
$s$: Suction/injection parameter (–)
